# Single-cell transcriptome analysis profiles cellular dynamics and transcriptional changes in diabetic wound tissues following ESWT treatment

**DOI:** 10.3389/fphys.2025.1693937

**Published:** 2025-11-17

**Authors:** Dongyu Li, Yu Wang, Yunlong Wang, Changhai Shao, Lei Wang, Shijie Xin, Yuewen Ma

**Affiliations:** 1 Department of VIP In-Patient Ward, The First Hospital of China Medical University, Shenyang, Liaoning, China; 2 Department of Rehabilitation, The First Hospital of China Medical University, Shenyang, Liaoning, China; 3 Department of Thoracic Surgery, Angang General Hospital, Anshan, Liaoning, China; 4 Department of Vascular and Thyroid Surgery, the First Hospital of China Medical University, Shenyang, Liaoning, China

**Keywords:** diabetic wound, extracorporeal shock wave therapy, single cell RNA sequencing, endothelial cells, microenvironment remodeling

## Abstract

**Introduction:**

Diabetic wounds (DWs) remain a major complication of diabetes mellitus, characterized by impaired healing and limited therapeutic options. Extracorporeal shock wave therapy (ESWT), a non-invasive physical modality, has recently shown promise in accelerating chronic wound repair, yet the underlying cellular mechanisms remain poorly understood.

**Methods:**

Here, we employed single-cell RNA sequencing (scRNA-seq) to construct a comprehensive cellular atlas of DW tissues treated with ESWT, profiling approximately 39,475 cells.

**Results:**

Our analysis identified 12 major cell populations, including macrophages, fibroblasts, endothelial cells, keratinocytes, and immune subsets, and revealed widespread transcriptional reprogramming associated with ESWT treatment. ESWT promoted the expansion of reparative macrophages, activated proregenerative fibroblast states, and restored angiogenic programs in endothelial cells. Moreover, cell–cell communication analysis revealed that ESWT not only attenuates pro-inflammatory signaling but also activates immune cell communication networks, thereby enhancing T cell, NK cell, and dendritic cell interactions. These changes collectively promote immune regulation and tissue repair, contributing to the restoration of a balanced wound microenvironment.

**Discussion:**

Together, these findings provide a high-resolution single-cell map of ESWT mediated cellular and molecular alterations in DWs and uncover key cellular pathways contributing to improved tissue repair. This study offers new insights into the mechanisms of ESWT and supports its translational potential as a therapeutic strategy for chronic wound management.

## Introduction

Diabetic wounds (DW) represent a severe chronic complication of diabetes, characterized by impaired and delayed healing under hyperglycemic conditions (Patel et al., 2019). This condition imposes a substantial socioeconomic burden and profoundly diminishes patients’ quality of life (Berthiaume and Hsia, 2022). Despite the availability of standard treatments aimed at controlling blood glucose and infection, a considerable proportion of patients remain at high residual risk, often progressing to adverse clinical outcomes (Shi et al., 2024). Thus, there is an urgent need to develop non-surgical strategies that actively enhance wound repair and improve patient prognosis.

Extracorporeal shock wave therapy (ESWT), a non-invasive intervention, has been extensively employed in the management of musculoskeletal disorders (Charles et al., 2023), cartilage and subchondral bone disease (An et al., 2020) and urinary stone disease (Skov-Jeppesen et al., 2023). The underlying principles of ESWT are rooted in mechanotransduction—the process by which cells convert mechanical stimuli into biochemical signals. This triggers a range of cellular responses, including the activation of signaling pathways such as VEGF, PI3K/Akt, and AMPK, which are crucial for promoting angiogenesis (Lv et al., 2023), modulating inflammation (Medina, 2023), stimulating tissue regeneration and stem cell recruitment (Jin et al., 2025). Clinically, ESWT has been approved for the treatment of various musculoskeletal conditions and is increasingly applied in wound care. Recent clinical studies further suggested that ESWT significantly accelerated chronic wound closure (Wu et al., 2024).

Multiple randomized controlled trials and meta-analyses have demonstrated that ESWT significantly improves the healing rate of chronic diabetic foot ulcers (DFUs), reducing wound size and time to closure compared to standard care alone. In preclinical studies, animal models of DW healing have consistently shown that ESWT accelerates re-epithelialization, granulation tissue formation, and overall wound closure. Its efficacy is attributed to its multi-faceted mechanisms of action. Firstly, it induces neovascularization by upregulating pro-angiogenic factors, thereby enhancing blood flow to the ischemic wound bed. Secondly, it possesses potent anti-inflammatory and immunomodulatory effects, promoting the transition from a pro-inflammatory M1 to a pro-healing M2 macrophage phenotype. Furthermore, ESWT has been shown to enhance fibroblast proliferation and collagen synthesis, thereby facilitating extracellular matrix remodeling. However, the precise cellular and molecular mechanisms underlying the therapeutic benefits of ESWT in DW remain poorly understood.

Single-cell RNA sequencing (scRNA-seq) provides a powerful platform to delineate cellular heterogeneity and to capture dynamic transcriptional alterations at single-cell resolution (Cheng et al., 2023; Chen et al., 2023; Cui et al., 2025). In this study, we employed scRNA-seq to generate a comprehensive atlas of cell populations and transcriptional programs in DW tissues subjected to ESWT. We characterized both immune and non-immune compartments, revealed dynamic cellular shifts in response to therapy, and identified candidate therapeutic cell subsets potentially mediating ESWT-driven tissue repair. Collectively, our findings offer novel mechanistic insights into ESWT-induced DW healing and may inform the development of more effective therapeutic strategies for this challenging complication.

## Materials and methods

### Establishment of a Streptozotocin (STZ)-Induced DW model and ESWT treatment

A total of 12 male Sprague–Dawley rats (8 weeks old, weighing 260–280 g) were obtained from GemPharmatech Co., Ltd. The experimental protocol was approved by the Ethics Committee on the Care and Use of Laboratory Animals at China Medical University (Ethics No. CMU20251368). All animals were housed under specific pathogen-free (SPF) and comfortable conditions (20 °C ± 2 °C, 12-h light/dark cycle, 50% ± 5% relative humidity) with free access to food and water. After a 7-day adpative period, the rats were randomly assigned to four groups: control, DW, ESWT 7d and ESWT 14d.

Experimental diabetes was induced in rats of the DW and ESWT groups by a single intraperitoneal injection of streptozotocin (STZ, 65 mg/kg) freshly prepared in citrate buffer (pH 4.5), as previously described (Zhang et al., 2024). Rats in the control group received an equal volume of citrate buffer alone. Blood glucose levels were monitored every 2 days. Rats with fasting blood glucose ≥16.7 mmol/L on three consecutive measurements were considered diabetic and included in subsequent experiments. For animals in which the initial injection failed to induce hyperglycemia (fasting glucose <16.7 mmol/L), a booster dose of STZ (30 mg/kg) was administered 5–7 days later, with only one repeat injection permitted to minimize toxicity. To maintain a stable diabetic state and prevent acute complications, low-dose insulin (1–2 IU) was administered as needed when blood glucose exceeded 33.3 mmol/L or when clinical signs of distress were observed. Animals with persistent hypoglycemia (<16.7 mmol/L) or severe systemic illness were excluded. At day 21 after diabetes induction, a 2.5 cm × 2.5 cm full-thickness excisional wound was created on the dorsal skin of each rat.

ESWT was performed using the ROLAND2 EXPERT device (PAGANI Elettronica, Italy) on days 24 and 27. Shock waves were applied at an energy flux density of 0.12 mJ/mm^2^ with a frequency of 3 Hz, delivering 100 pulses per centimeter of wound length, as previously described (Yang et al., 2011). Wound healing was monitored and documented by digital photography throughout the experimental period. All animals were euthanized with 5% isoflurane and samples were harvested on day 28 for the ESWT 7d group, and on day 35 for the control, DW, and ESWT 14d groups. Each wound sample was carefully excised to include both the wound edge and base, with an additional 2–3 mm margin of surrounding tissue to ensure capture of relevant healing zones. The collected tissues were evenly divided: one portion was processed for scRNA-seq, and the other portion was fixed and embedded for histological examination. For scRNA-seq, fresh tissues were immediately subjected to enzymatic digestion to prepare single-cell suspensions according to standard protocols.

### Single-cell suspension preparation and 10× genomics library generation

Wound tissues were collected and immediately preserved in sterile tissue storage solution containing 10 mL of 1× Dulbecco’s Phosphate-Buffered Saline (DPBS; Thermo Fisher, Cat. no. 14190144). Tissue digestion was performed using 0.25% Trypsin (Thermo Fisher, Cat. no. 25200-072) supplemented with 10 μg/mL DNase I (Sigma, Cat. no. 11284932001), dissolved in PBS containing 5% fetal bovine serum (FBS; Thermo Fisher, Cat. no. SV30087.02). Samples were incubated at 37 °C with gentle agitation, and dissociated cells were collected every 20 min. The resulting cell suspensions were sequentially filtered to remove debris, followed by red blood cell lysis using 1× RBC Lysis Solution (Thermo Fisher, Cat. no. 00-4333-57). Cell viability was assessed using the Countess® II Automated Cell Counter (Thermo Fisher). High-quality single-cell suspensions were then submitted to Majorbio Bio-pharm Technology Co., Ltd. (Shanghai, China) for downstream single-cell RNA sequencing (scRNA-seq). Single-cell libraries were prepared following the manufacturer’s standard protocol using the Chromium Single Cell 3′ Solution (10× Genomics, Pleasanton, CA, United States). Briefly, cell suspensions were loaded onto the Chromium Controller to generate Gel Beads-in-Emulsion (GEMs), ensuring near-saturation capture of individual cells with uniquely barcoded beads containing unique molecular identifiers (UMIs). After reverse transcription, cDNA was synthesized, amplified, and subjected to adaptor ligation and library construction. Final sequencing libraries were quantified and sequenced on an Illumina NovaSeq X Plus platform (paired-end, PE150 mode).

### scRNA-seq data processing and quality control

To investigate cellular heterogeneity in DW tissues following ESWT treatment, scRNA-seq was performed using the 10× Genomics Chromium platform. Raw sequencing data were processed with the Seurat R package to generate Seurat objects and perform quality control. To ensure high-quality and biologically meaningful data, stringent filtering criteria were applied to remove low-quality cells, doublets, and dying cells. Cells were retained if they met the following thresholds: Number of detected genes (nFeature_RNA): 300–7,000, to exclude low-complexity cells and potential multiplets; Mitochondrial gene percentage (mt_percent): <10%, to remove stressed or dying cells; Hemoglobin gene percentage (HB_percent): <3%, to minimize contamination from lysed red blood cells; Total UMI counts (nCount_RNA): between 1,000 and the 97th percentile of each dataset, to exclude extreme outliers and potential doublets. These thresholds were determined based on the distribution of QC metrics across all samples and were consistent with published filtering practices for complex tissue single-cell datasets. Following QC, the data were normalized using the NormalizeData function in Seurat. Batch effects across samples were corrected using the Harmony integration algorithm. Finally, dimensionality reduction and visualization of cell clusters were performed using the uniform manifold approximation and projection (UMAP) method.

### Identification of differentially expressed genes (DEGs) and functional enrichment analysis

DEGs were identified using the FindAllMarkers function in Seurat with default parameters. Genes were considered significant if they satisfied both an adjusted p-value < 0.05 and an absolute log2 fold change (|log2FC|) > 0.25. Statistical testing was performed using the default Wilcoxon rank-sum test. To investigate the biological functions associated with DEGs across different cell subpopulations, Gene Ontology (GO) and Kyoto Encyclopedia of Genes and Genomes (KEGG) enrichment analysis was performed using the clusterProfiler R package, and results were visualized with ggplot2.

### Pseudotime trajectory and CytoTRACE analysis

To explore the temporal dynamics of cellular states within the DW microenvironment following ESWT treatment, pseudotime trajectory inference was conducted using the Monocle2 algorithm. For each selected cell type, Seurat objects were converted into Monocle2 CellDataSet objects using the SeuratWrappers function. Highly variable genes identified in Seurat were used for ordering cells along the trajectory. Dimensionality reduction was carried out using the DDRTree algorithm, which embeds cells in a low-dimensional space while preserving potential branching structures. Cells were ordered along the inferred pseudotime axis using the orderCells function, enabling visualization of cell state transitions and branching events. DEGs across pseudotime and branch points were identified using the differentialGeneTest function with the *q*-value threshold set at 0.01. Branch-dependent gene expression programs were further explored using the Branch Expression Analysis Modeling (BEAM) algorithm to identify genes that distinguish alternative cell fate decisions. Trajectory plots and branch-dependent heatmaps were generated to visualize transcriptional dynamics. These analyses allowed reconstruction of the temporal evolution of cellular states and identification of key regulators involved in ESWT-mediated DW healing.

### Cell–cell communication analysis using CellChat

Intercellular communication within the DW microenvironment was assessed using the CellChat R package. To construct communication networks, signaling genes expressed in at least 10% of cells within a given group were retained, while low-quality genes and cells were excluded. Overexpressed ligands, receptors, and ligand–receptor interactions were identified using the default pipeline. The resulting communication networks were visualized with circle plots, and differences in interaction strength and signaling patterns were quantified between groups. This analysis enabled the identification of dynamic changes in intercellular communication associated with ESWT treatment and highlighted key pathways underlying the wound healing process.

### Histological staining

Collected wound tissues were fixed in 4% paraformaldehyde, followed by graded ethanol dehydration and xylene clearing. Samples were subsequently embedded in paraffin, and sections of 4 μm thickness were prepared using a microtome. Hematoxylin and eosin (H&E) and Masson’s trichrome staining were performed to evaluate histological alterations in DW tissues after ESWT treatment. The sectioning and staining procedures were carried out by Nanjing Youmeng Biotechnology Co., Ltd.

### Statistical analysis

All statistical analyses were conducted in R software unless otherwise specified. For scRNA-seq data, preprocessing, dimensionality reduction, clustering, and differential expression analyses were performed using the Seurat package. Cell types were annotated based on canonical marker genes and confirmed by manual curation. DEGs between groups or clusters were identified using the Wilcoxon rank-sum test, with significance thresholds set at adjusted *p* < 0.05 and |log_2_ fold change| > 0.25.

## Results

### ESWT promoted wound closure and tissue regeneration in diabetic rats

To evaluate the therapeutic effects of ESWT on DW healing, a full-thickness excisional wound model was established in diabetic rats and treated with or without ESWT. As shown in Supplementary Figure S1A, wound closure was markedly delayed in the DW group compared with the control group, whereas ESWT treatment significantly accelerated wound contraction and epithelial regeneration from day 7 onward. By day 21, most wounds in the ESWT group were almost completely healed, showing a smooth epithelial surface comparable to that of the control group.

Histological analysis further confirmed the beneficial effects of ESWT on wound repair. Hematoxylin and eosin (HE) staining revealed an evident re-epithelialization and restoration of skin appendages in the ESWT group, whereas the DW group exhibited incomplete epithelial coverage and disorganized tissue structure (Supplementary Figure S1B). Masson’s trichrome staining showed increased collagen deposition and better collagen fiber alignment in the ESWT group compared with the DW group, indicating enhanced extracellular matrix remodeling. Quantitative analysis demonstrated that ESWT significantly improved the wound healing rate (Supplementary Figure S1C) and increased both the re-epithelialization rate and collagen deposition rate (Supplementary Figure S1D). Collectively, these findings indicate that ESWT markedly promotes wound closure, epithelial regeneration, and matrix remodeling in diabetic wounds.

### scRNA-seq profiling uncovered cellular heterogeneity in DW microenvironment following ESWT treatment

**FIGURE 1:**
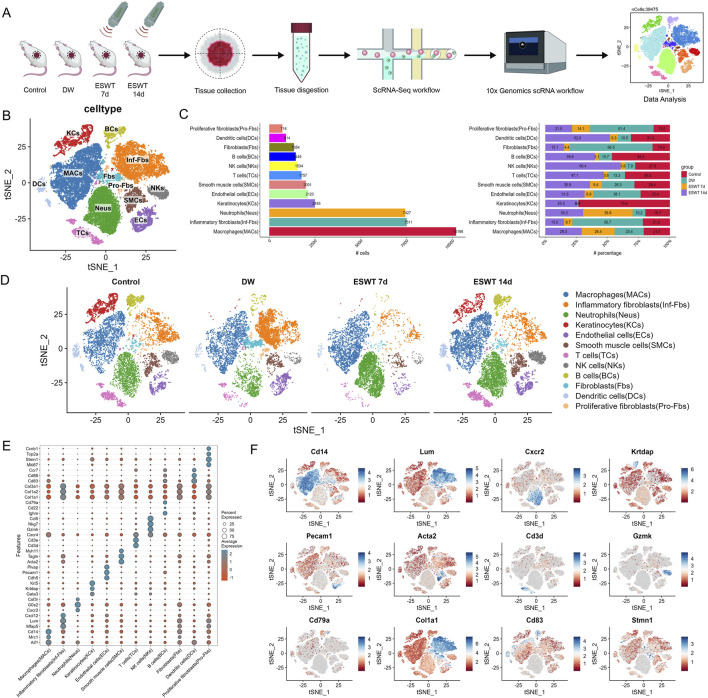
ESWT reshaped the cellular landscape of DWs revealed by single-cell RNA sequencing. **(A)** Schematic diagram of the experimental workflow. Wound tissues from control, DW, ESWT 7d, and ESWT 14d groups were collected, dissociated, and subjected to 10x Genomics scRNA-seq. Data analysis identified distinct cellular populations. **(B)** t-SNE plot showing clustering of major cell populations, including macrophages (MACs), inflammatory fibroblasts (Inf-Fbs), neutrophils (Neus), keratinocytes (KCs), endothelial cells (ECs), smooth muscle cells (SMCs), T cells (TCs), NK cells (NKs), B cells (BCs), fibroblasts (Fbs), dendritic cells (DCs), and proliferative fibroblasts (Pro-Fbs). **(C)** Bar plots depicting the number (left) and relative proportion (right) of each cell type across different groups, highlighting dynamic remodeling of cellular composition during ESWT treatment. **(D)** t-SNE visualization of cell type distributions in control, DW, ESWT 7d, and ESWT 14d groups, demonstrating marked shifts in immune and stromal cell populations upon ESWT treatment. **(E)** Dot plot showing expression patterns of canonical marker genes used for cell type annotation, with dot size representing the percentage of expressing cells and color intensity indicating average expression level. **(F)** Feature plots illustrating representative marker gene expression for different cell populations: Cd14 (macrophages), Lum (fibroblasts), Cxcr2 (neutrophils), Krtdap (keratinocytes), Pecam1 (endothelial cells), Acta2 (smooth muscle cells), Cd3d (T cells), Gzmk (NK cells), Cd79a (B cells), Col1a1 (fibroblasts), Cd83 (dendritic cells), and Stmn1 (proliferative fibroblasts).

To gain insights into the cellular dynamics underlying ESWT-induced wound repair, scRNA-seq was performed on unsorted cells obtained from control tissues and DW tissues subjected to ESWT or left untreated. The workflow of this study was shown in Figure 1A. After quality control, a total of 39,475 cells were retained for downstream analysis, including 10,418 from control group, 10,622 cells from the DW group, 6,882 from ESWT 7d, and 11,553 from ESWT 14d. Quality control metrics were presented in Supplementary Figures S2A,B. Unsupervised clustering and tSNE visualization revealed four major cell lineages, including immune cells, stromal cells, epithelial cells, and endothelial cells (Supplementary Figure S2C). The expression of canonical marker genes, such as Ptprc (immune cells), Col1a1 (stromal cells), Krt5 (epithelial cells), and Pecam1 (endothelial cells), confirmed accurate cell-type annotation (Supplementary Figure S2D). Comparative analysis of cellular composition demonstrated distinct shifts in cell population dynamics following ESWT treatment (Supplementary Figure S2E).

To further resolve cellular heterogeneity within these four major lineages, we performed high-resolution clustering. Unbiased clustering revealed 12 major cell types with distinct expression patterns. As shown in Figure 1B, unsupervised clustering and tSNE visualization revealed 12 major cell populations across all groups, including macrophages (MACs), inflammatory fibroblasts (Inf-Fbs), neutrophils (Neus), keratinocytes (KCs), endothelial cells (ECs), smooth muscle cells (SMCs), T cells (TCs), NK cells (NKs), B cells (BCs), fibroblasts (Fbs), dendritic cells (DCs), and proliferative fibroblasts (Pro-Fbs). In control skin tissues, KCs were abundant with moderate stromal cells and moderate immune cells (Figure 1C). In DW group, Inf-Fbs, Fbs and Neus markedly expanded and MACs dominated, whereas KCs were nearly absent, indicating an inflammation-skewed state with impaired epithelium and vasculature. By ESWT 7d, inflammatory compartments (Inf-Fbs) and stromal cells began to contract, with a noticeable rise of adaptive immune cells (Neus and MACs); KCs remained low. By ESWT 14d, KCs rebounded prominently, Pro-Fbs and immune cells further increased, and ECs, SMCs were restored. This suggested that ESWT dynamically modulated the immune microenvironment during DW healing and significantly promoted re-epithelialization during the later stages of wound healing (Figure 1D). Dot plot analysis confirmed cell-type-specific expression of canonical markers across 12 clusters (Figure 1E). To validate cell type annotation, we visualized the expression patterns of representative marker genes across the tSNE space (Figure 1F). Feature plots of canonical markers further confirmed the spatial localization of corresponding cell types across the tSNE map.

### ESWT treatment altered macrophage polarization and heterogeneity in DW tissues

**FIGURE 2:**
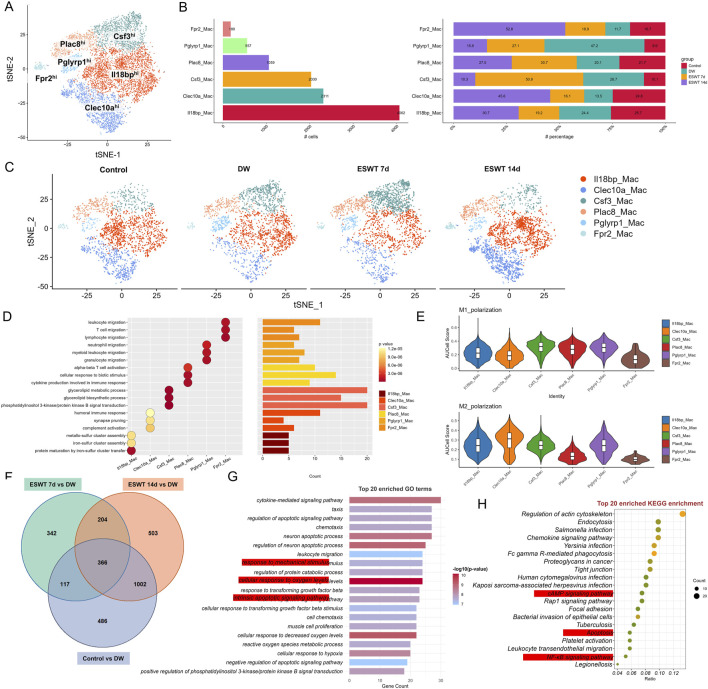
ESWT remodeled macrophage subclusters and promoted M2 polarization in DWs. **(A)** t-SNE visualization showing six macrophage subclusters identified by marker gene expression. **(B)** Bar plots showing the cell numbers (left) and relative proportions (right) of each macrophage subcluster across control, DW, ESWT 7d, and ESWT 14d groups, demonstrating dynamic remodeling under ESWT treatment. **(C)** t-SNE plots illustrating the distribution of macrophage subclusters in each group. **(D)** Functional enrichment analysis of subcluster marker genes. **(E)** Violin plots of AUCell scores for M1-polarization and M2-polarization related gene signatures across macrophage subclusters, indicating ESWT-induced enhancement of M2 polarization, particularly in Clec10a_Mac. **(F)** Venn diagram of DEGs between control, DW, ESWT 7d, and ESWT 14d groups, with a large number of overlapping genes reflecting shared ESWT responses. **(G)** Top 20 enriched GO terms for intersected DEGs, mainly involving immune regulation, inflammatory signaling, and oxidative stress response. **(H)** Top 20 enriched KEGG pathways, including actin cytoskeleton regulation, chemokine signaling, FcγR-mediated phagocytosis, NF-κB signaling, and AMPK signaling, suggesting mechanistic pathways through which ESWT modulates macrophage function and promotes wound repair.

To investigate the impact of ESWT on macrophage subpopulations in DW tissues, we conducted scRNA-seq on wound tissues from non-healing and ESWT-treated groups at different time points. Unbiased clustering revealed the presence of 6 macrophage subpopulations, which were further classified based on their distinct gene expression profiles (Figure 2A). Analysis of macrophage subpopulations revealed distinct distribution patterns across different groups (Figure 2B). The t-SNE plots showed a clear shift in macrophage population distribution across different groups over time (Figure 2C). Among the six identified subclusters, Il18bp^hi^ Macs were the most abundant, whereas Fpr2^hi^ Macs were the least represented (180 cells, mainly in the ESWT 14d group). Proportionally, ESWT treatment induced notable shifts in subpopulation composition. Among the six identified macrophage subclusters, Clec10a^hi^ accounted for a total of 2311 cells and showed a distinct response to ESWT treatment. In the DW group, Clec10a^hi^ exhibited the lowest proportion (13.5%), whereas ESWT markedly increased its abundance, reaching 16.1% in ESWT 7d and peaking at 45.6% in ESWT 14d. This time-dependent enrichment suggested that ESWT progressively promoted the expansion of Clec10a^hi^ Macs during wound healing. As shown in Figure 2D, the Il18bp^hi^ cluster was significantly enriched in pathways related to iron–sulfur cluster assembly and protein maturation, suggesting a potential role in metabolic regulation and redox homeostasis. The Clec10a^hi^ subset showed enrichment in complement activation and humoral immune response, indicating its involvement in innate immune regulation and clearance of cellular debris. The Csf3^hi^ population was enriched for synapse pruning and phosphatidylinositol 3-kinase/protein kinase B (PI3K-Akt) signaling, implying a role in intercellular communication and signal transduction during tissue remodeling. The Plac8^hi^ cluster exhibited enrichment in cytokine production, cellular response to biotic stimulus, and T-cell activation, indicating a pro-inflammatory and immune-stimulatory phenotype, potentially representing activated macrophages during the early inflammatory phase of wound healing. In contrast, the Pglyrp1^hi^ subset was associated with leukocyte and neutrophil migration and myeloid cell chemotaxis, suggesting a function in immune cell recruitment and inflammation resolution. Finally, the Fpr2^hi^ cluster was enriched in lymphocyte migration and alpha–beta T-cell activation, implying an immunomodulatory role that may facilitate macrophage–lymphocyte crosstalk and promote wound resolution. AUCell scoring showed heterogeneous polarization patterns across subpopulations: Il18bp^hi^ Macs and Pglyrp1^hi^ Macs displayed higher M1 polarization scores, whereas Clec10a^hi^ Macs and Pglyrp1^hi^ Macs exhibited elevated M2 polarization scores, suggesting distinct pro-inflammatory and pro-repair functional states within the macrophage compartment (Figure 2E).

To identify genes that were consistently differentially expressed across the different time points of ESWT treatment in DW tissues, we performed an intersection analysis of the DEGs between control vs. DW and ESWT 7d, 14d vs. DW. A total of 366 intersected genes were collected and the GO enrichment analysis for the 366 intersecting genes was shown in the Figure 2F. GO enrichment analysis of the intersection genes revealed significant enrichment in immune-related biological processes, including response to mechanical stimulus, leukocyte migration, regulation of cytokine production, neutrophil chemotaxis, and T cell activation, as well as processes related to actin cytoskeleton organization and phagocytosis, indicating their potential roles in immune cell recruitment and activation during wound repair (Figure 2G).

Among the most significantly enriched GO-BP terms were positive regulation of apoptotic signaling pathway, regulation of apoptotic signaling pathway, cellular response to hypoxia and response to mechanical stimulus. These processes suggested that ESWT modulated cellular mechanisms involved in mechanotransduction, reduced apoptosis, enhanced the cellular response to hypoxia, and helped cells adapt to the hypoxic environment of diabetic wounds, thereby promoting cell survival, proliferation, and efficient tissue remodeling. KEGG pathway analysis further demonstrated that these genes were mainly involved in regulation of actin cytoskeleton, endocytosis, chemokine signaling pathway, Fc gamma R-mediated phagocytosis, tight junction, and leukocyte transendothelial migration, along with key signaling cascades such as cAMP, Rap1, and NF-κB signaling pathways (Figure 2H). These findings suggested that the intersection genes might contribute to ESWT-mediated DW healing through enhancing immune cell trafficking, strengthening barrier integrity, and activating pro-healing signaling networks.

**FIGURE 3:**
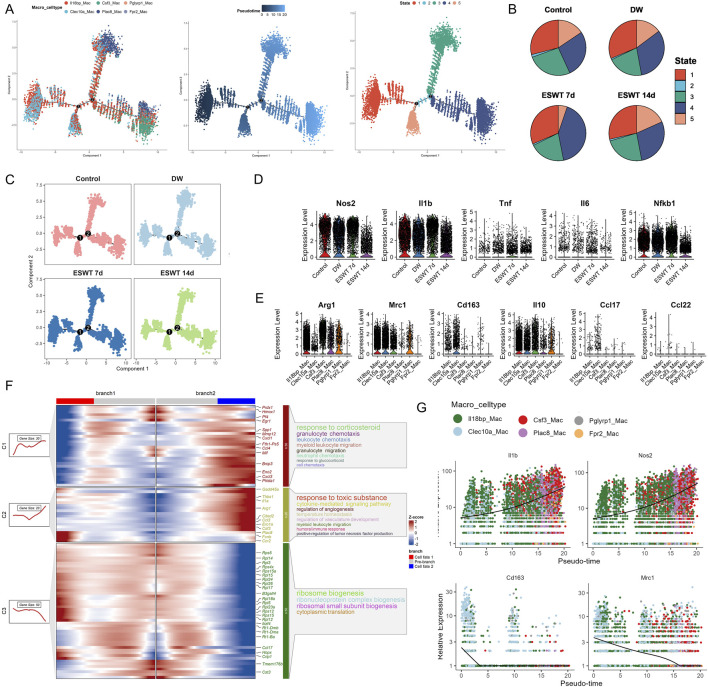
ESWT promoted macrophage phenotypic transition and alleviated inflammatory responses in DWs. **(A)** Pseudotime trajectory analysis of macrophage subclusters (Il18bp_Mac, Clec10a_Mac, Csf3_Mac, Plac8_Mac, Pglyrp1_Mac, Frp2_Mac). Cells were ordered along developmental trajectories, with pseudotime (middle) and distinct state assignments (right) indicating dynamic differentiation. **(B)** Pie charts showing the distribution of pseudotime states in control, DW, ESWT 7d, and ESWT 14d groups, highlighting ESWT-induced remodeling of macrophage states. **(C)** Pseudotime distribution of macrophages across experimental groups, demonstrating that ESWT treatment shifted macrophages toward later pseudotime states associated with resolution and repair. **(D)** Violin plots of representative M1 pro-inflammatory genes (Nos2, Il1b, Tnf, Il6, Nfkb1) showing downregulation after ESWT treatment. **(E)** Violin plots of representative M2 anti-inflammatory and reparative genes (Arg1, Mrc1, Cd163, Il10, Ccl17, Ccl22) showing upregulation under ESWT, indicating enhanced M2 polarization. **(F)** Branched heatmap of pseudotime-dependent gene expression modules. Genes in cluster 1 were enriched in inflammatory and chemotaxis pathways, whereas cluster 2 and 3 genes were associated with angiogenesis, ribosome biogenesis, and tissue repair functions. **(G)** Smoothed pseudotime expression trends of key genes (Il1b, Nos2, Cd163, Mrc1) across macrophage subclusters, confirming that ESWT reduced inflammatory gene expression while promoting M2-associated markers.

The Monocle2 analysis was performed to investigate the pseudotime trajectory of macrophage. The clustering of macrophages showed clear separation into different states along the trajectory. Monocle2 trajectory analysis revealed a continuous developmental landscape of macrophage subpopulations, arranged along distinct pseudotime branches (Figure 3A). Six macrophage subclusters were distributed across five transcriptional states. Pseudotime ordering suggested a progression from State 1, dominated by Il18bp^hi^ Macs and Clec10a^hi^ Macs, toward later states enriched in Csf3^hi^ Macs, Plac8^hi^ Macs, and Fpr2^hi^ Macs (Figure 3B). As shown in Figure 3C, state composition differed markedly among experimental groups. In the DW group, State 1 was predominant, whereas ESWT treatment shifted the distribution toward States 4 and 5. Notably, macrophages from the ESWT 7-day group were mainly enriched in State 4, suggesting that ESWT promoted the transition of macrophages toward a pro-healing phenotype at this intermediate stage of differentiation. These findings indicated that ESWT induced a temporal reprogramming of macrophage states, thereby accelerating the progression toward reparative phenotypes along the differentiation trajectory.

Then we investigated the effect of ESWT on M1 macrophage polarization markers during the treatment period. Our findings indicated that the expression levels of Cd68, Cd86, Il1b, Tnf, and Nos2 were significantly modulated by ESWT treatment, with ESWT group showing decreased expression of pro-inflammatory markers compared to DW group (Figures 3D,E). By analyzing gene expression along these trajectories, the BEAM method helped identify key regulators and pathways involved in macrophage differentiation. The BEAM analysis of fate 2 revealed significant gene expression changes across the two branches, as visualized in the heatmap (Figure 3F). Branch 1 was predominantly associated with the response to corticosteroid, including genes involved in granulocyte chemotaxis and neutrophil chemotaxis such as Prdx1, Mmp12, and Cxcl1, indicating an active immune response and cellular migration processes. On the other hand, Branch 2 showed a clear enrichment in pathways related to response to toxic substances, including cytokine-mediated signaling pathways, angiogenesis, and myeloid leukocyte migration. Key genes such as Gadd45a, Thbs1, and Cited2 were differentially expressed in this branch, suggesting a role in tissue remodeling and response to environmental stress. Additionally, ribosome biogenesis was notably enriched across the branches, with genes like Rps5, Rpl12, and Rps15 showing consistent expression patterns. These results highlighted distinct regulatory networks driving macrophage differentiation and activation along the fate 2 trajectory, with specific pathways governing immune response and cellular biosynthesis processes.

Interestingly, M1 macrophage polarization markers Il1b and Nos2 showed an increase in expression along pseudotime, with higher expression observed in cells at later stages (red and green clusters). This finding was consistent with the activation of inflammatory and pro-inflammatory pathways in DW microenvoirment (Figure 3G). Consistently, M2 macrophage polarization markers Cd163 and Mrc1 exhibited a distinct expression pattern with higher expression at early pseudotime stages, especially in Clec10a^hi^ and Il18bp^hi^ cluster, followed by a sharp decrease in the later stages. The expression dynamics in these clusters suggested that Clec10a^hi^ and Il18bp^hi^ Mac might represent crucial macrophage subtypes influenced by ESWT, potentially driving the polarization of macrophages toward an anti-inflammatory or reparative phenotype. Our findings demonstrated that ESWT markedly reshaped macrophage heterogeneity in DWs, inducing time-dependent shifts in subpopulation composition and transcriptional states. Specifically, ESWT promoted the expansion of reparative subclusters and accelerated the transition toward pro-healing trajectories.

### ESWT reshaped fibroblast heterogeneity by enriching pro-repair subclusters in DW tissues

**FIGURE 4:**
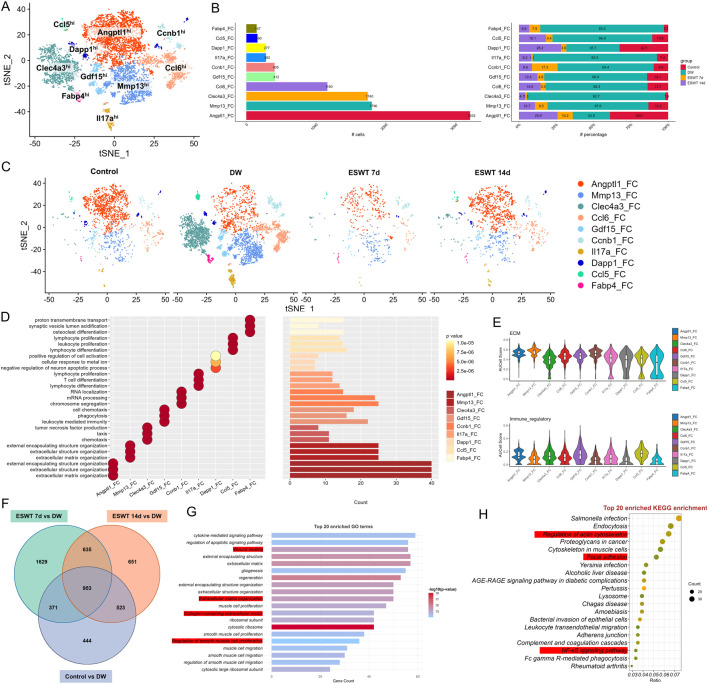
ESWT reshaped fibroblast subclusters and enhanced ECM remodeling in DWs. **(A)** t-SNE plot showing eight fibroblast subclusters identified by marker genes. **(B)** Bar plots displaying the absolute numbers (left) and relative proportions (right) of each fibroblast subcluster in control, DW, ESWT 7d, and ESWT 14d groups, demonstrating that ESWT progressively restored fibroblast heterogeneity. **(C)** t-SNE visualization of fibroblast distributions across groups, showing expansion of Angptl1_FC and reduction of pathological fibroblast subsets (Mmp13_FC, Clec4a3_FC) after ESWT treatment. **(D)** Functional enrichment analysis of fibroblast subclusters, with enriched GO terms related to ECM organization, collagen metabolic process, and wound healing. **(E)** Violin plots of AUCell scores for ECM-related (top) and immune-regulatory (bottom) gene signatures across fibroblast subclusters, indicating that ESWT enhanced reparative ECM activity while reducing inflammatory fibroblast phenotypes. **(F)** Venn diagram of DEGs between control, ESWT 7d, ESWT 14d and DW groups, showing a substantial set of overlapping genes regulated by ESWT. **(G)** Top 20 enriched GO terms for DEGs, highlighting ECM organization, collagen fibril assembly, and regulation of angiogenesis. **(H)** Top 20 enriched KEGG pathways for DEGs, including AGE-RAGE signaling, ECM–receptor interaction, focal adhesion, PI3K-Akt signaling, and NF-κB signaling, suggesting that ESWT regulates fibroblast function through key extracellular and inflammatory pathways.

To assess how ESWT modulated fibroblast subpopulations in DW tissues, we performed scRNA-seq on DW samples from different groups across multiple time points. As shown in Figure 4A, tSNE analysis revealed nine distinct fibroblast subclusters characterized by high expression of representative marker genes. Among them, Angptl1^hi^ and Mmp13^hi^ Fbs were the most abundant populations, followed by Clec4a3^hi^ and Ccl6^hi^ Fbs. Cell proportion analysis showed marked differences in fibroblast composition across groups (Figure 4B). In DW, Angptl1^hi^ Fbs were markedly reduced compared with control group, whereas ESWT treatment significantly increased their proportion, particularly at 14 days, where they reached the highest level. Mmp13^hi^, Clec4a3^hi^, and Ccl6^hi^ subclusters also showed partial recovery after ESWT (Figure 4C). Conversely, Fabp4^hi^, Ccl5^hi^, and Dapp1^hi^ subclusters remained at low abundance in all groups, while Gdf15^hi^ and Ccnb1^hi^ Fbs exhibited moderate ESWT-induced changes. GO enrichment analysis of fibroblast subcluster marker genes revealed distinct functional specializations (Figure 4D). Angptl1^hi^ and Mmp13^hi^ Fbs were enriched in extracellular matrix organization and structure formation, suggesting roles in tissue remodeling. Clec4a3^hi^ and Gdf15^hi^ Fbs were associated with immune regulation, including leukocyte proliferation, lymphocyte activation, and TNF production, indicating potential involvement in immunomodulation and inflammatory responses during wound healing. Ccl5^hi^ Fbs were linked to lymphocyte and T cell differentiation and chemokine-mediated immune responses, while Il17a^hi^ and Dapp1^hi^ Fbs were related to inflammatory signaling and cell activation processes. AUCell-based functional scoring revealed that fibroblast subclusters exhibited distinct phenotypic specializations (Figure 4E). For the ECM remodeling signature, Angptl1^hi^, Mmp13^hi^, Clec4a3^hi^, and Ccl6^hi^ subclusters displayed the highest activity scores, indicating their predominant roles in extracellular matrix organization and tissue structural remodeling. In the immune-regulatory signature, Angptl1^hi^, Clec4a3^hi^, Ccl6^hi^, and Gdf15^hi^ subclusters showed markedly elevated scores, suggesting their involvement in modulating immune cell responses and inflammatory resolution. These results highlight that ESWT-responsive fibroblast subsets might contribute to wound repair via distinct yet complementary functional programs—structural matrix rebuilding and immune regulation.

To further explore shared molecular changes induced by ESWT, we identified intersection genes from three comparisons: ESWT 7d vs. DW, ESWT 14d vs. DW, and Control vs. DW. Venn diagram analysis revealed 953 overlapping genes among the three contrasts, representing core ESWT-responsive transcripts in fibroblasts (Figure 4F). GO analysis of these intersection genes highlighted processes such as wound healing, extracellular matrix organization, cell adhesion and immune cell migrations, consistent with fibroblast-mediated repair functions (Figure 4G). KEGG pathway enrichment revealed significant associations with focal adhesion, regulation of actin cytoskeleton, PI3K-AKT and NF-κB signaling, leukocyte transendothelial migration, and AGE-RAGE signaling in diabetic complications (Figure 4H). These results indicated that ESWT might promote fibroblast-mediated tissue repair by enhancing ECM remodeling, cytoskeletal reorganization, and immune–vascular interactions. These pathways were critical in mediating inflammatory responses, enhancing ECM remodeling and cytoskeletal remodeling, all of which were vital for effective wound closure and regeneration.

**FIGURE 5:**
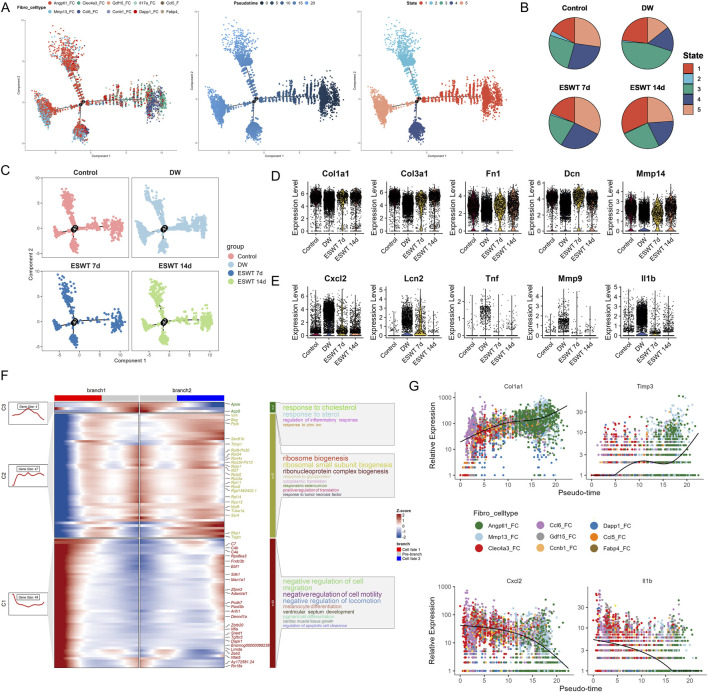
ESWT facilitated fibroblast phenotypic transition and promoted ECM remodeling during DW healing. **(A)** Pseudotime trajectory analysis of fibroblast subclusters. Cells were ordered along pseudotime (middle) and assigned into distinct states (right), indicating dynamic progression of fibroblast differentiation. **(B)** Pie charts showing pseudotime state distributions across control, DW, ESWT 7d, and ESWT 14d groups, demonstrating ESWT-driven remodeling of fibroblast states. **(C)** Pseudotime distribution plots highlighting the shift of fibroblasts in ESWT groups toward later pseudotime states associated with repair and ECM remodeling. **(D)** Violin plots showing expression levels of ECM-related genes (Col1a1, Col3a1, Fn1, Dcn, Mmp14), which were elevated after ESWT. **(E)** Violin plots of inflammatory and stress-related genes (Cxcl2, Lcn2, Tnf, Mmp9, Il1b), showing downregulation following ESWT treatment. **(F)** Branched heatmap illustrating pseudotime-dependent gene modules. Cluster 1 genes were enriched in cholesterol and immune response, cluster 2 in ribosome biogenesis, and cluster 3 in regulation of cell migration, suggesting functional divergence of fibroblast trajectories. **(G)** Smoothed pseudotime expression dynamics of representative genes (Col1a1, Timp3, Cxcl2, Il1b) across fibroblast subclusters, showing that ESWT promoted ECM-related gene expression while suppressing pro-inflammatory genes.

Monocle2 trajectory analysis revealed a branched differentiation pattern of fibroblast subclusters, with distinct distribution along pseudotime (Figure 5A). Notably, Angptl1^hi^ and Mmp13^hi^ subclusters were enriched in later pseudotime states, indicating potential involvement in the later phases of wound repair, including extracellular matrix remodeling and angiogenesis. In contrast, Clec4a3^hi^, Ccl6^hi^, and Gdf15^hi^ Fbs—identified as DW-associated fibroblast populations—were predominantly distributed in early-to-intermediate pseudotime states, indicating their persistence in non-healing conditions and potential involvement in sustaining inflammatory or dysregulated immune responses. State analysis showed that DW samples were enriched in intermediate states (States 3), while ESWT treatment, especially at 14 days, shifted fibroblast distribution toward late states (State 1), which may reflect progression toward a more reparative phenotype (Figure 5B).

Group-wise trajectory mapping further demonstrated that ESWT promoted the transition of fibroblasts along the differentiation continuum compared with DW, restoring a trajectory pattern closer to the Control group (Figure 5C). Subtype-specific trajectory plots confirmed that ESWT influenced pseudotime progression across multiple fibroblast phenotypes, suggesting a coordinated enhancement of both matrix reconstruction and immune–regulatory programs during wound healing. Group-wise differential expression analysis showed that key ECM remodeling genes, including Col1a1, Col3a1, Fn1, Dcn, and Mmp14, were significantly reduced in DW fibroblasts compared with Control, but were partially or fully restored following ESWT treatment, with the most pronounced upregulation observed at 14 days (Figure 5D). In contrast, inflammatory and immune-related markers such as Cxcl2, Lcn2, Tnf, Mmp9, and Il1b were markedly elevated in DW fibroblasts, indicating a persistent pro-inflammatory state. ESWT markedly suppressed the expression of these inflammatory genes, particularly by 14 days, suggesting an attenuation of pathological inflammation (Figure 5E). As shown in Figure 5F, GO enrichment of branch-specific genes revealed that branch 1 was associated with cholesterol/steroid response and ECM remodeling, while branch 2 was enriched in negative regulation of cell migration and inflammatory signaling. Pseudotime expression trends further confirmed that Col1a1 and Timp3 increased along the reparative trajectory, whereas Cxcl2 and Il1b declined (Figure 5G), highlighting a functional shift from inflammation toward structural reconstruction under ESWT intervention. Taken together, these results indicated that ESWT reshaped fibroblast heterogeneity in DW by selectively enriching pro-repair subclusters, suggesting enhanced extracellular matrix remodeling and angiogenesis potential during wound healing.

### ESWT drove phenotypic shift of neutrophils toward reparative and pro-angiogenic states in DW healing

**FIGURE 6:**
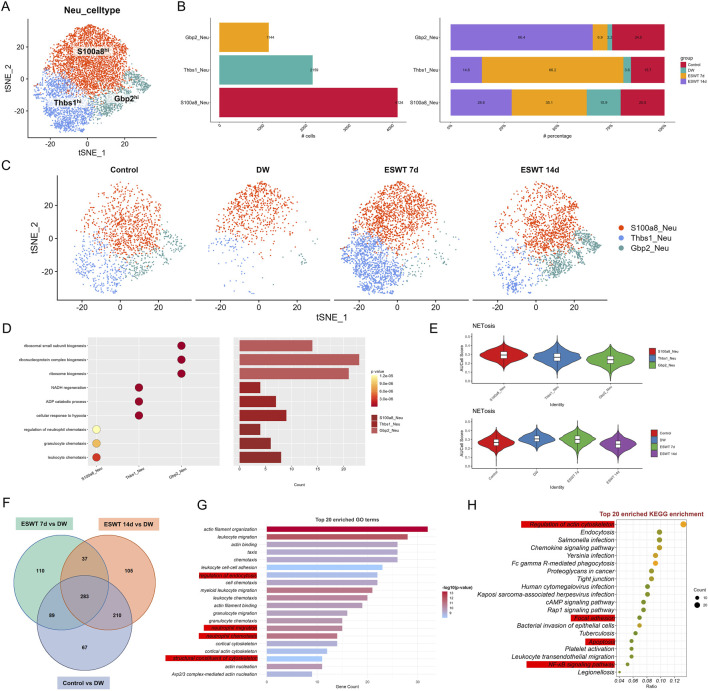
ESWT reshaped neutrophil subclusters and suppressed excessive NETosis in DWs. **(A)** t-SNE plot showing three neutrophil subclusters identified by marker genes. **(B)** Bar plots of absolute cell numbers (left) and relative proportions (right) of neutrophil subclusters across control, DW, ESWT 7d, and ESWT 14d groups, indicating ESWT-induced remodeling of neutrophil composition. **(C)** t-SNE visualization of neutrophil distributions in each group, demonstrating reduction of pathological S100a8_Neu and partial restoration of Gbp2_Neu and Thbs1_Neu after ESWT treatment. **(D)** Functional enrichment analysis of neutrophil subclusters, with GO terms enriched in leukocyte chemotaxis, inflammatory response, and granule secretion. **(E)** Violin plots of AUCell scores for NETosis-related gene signatures, showing that ESWT significantly reduced NETosis activity compared with DW. **(F)** Venn diagram of DEGs across control, DW, ESWT 7d, and ESWT 14d groups, showing both overlapping and ESWT-specific changes. **(G)** Top 20 enriched GO terms for DEGs, mainly related to cytoskeleton organization, chemotaxis, and neutrophil activation. **(H)** Top 20 enriched KEGG pathways for DEGs, including regulation of actin cytoskeleton, chemokine signaling, FcγR-mediated phagocytosis, NF-κB signaling, and bacterial infection pathways, suggesting that ESWT modulates neutrophil function through cytoskeletal and inflammatory signaling cascades.

Single-cell transcriptomic analysis identified three transcriptionally distinct neutrophil subpopulations in skin wound tissues: S100a8^hi^, Thbs1^hi^, and Gbp2^hi^ clusters (Figure 6A). Among them, S100a8^hi^ neutrophils constituted the largest proportion (4,124 cells), followed by Thbs1^hi^ (2,159 cells) and Gbp2^hi^ (1,144 cells) (Figure 6B). In the DW group, neutrophils were predominantly S100a8^hi^, indicating a sustained pro-inflammatory state (Figure 6C). ESWT treatment induced a marked shift in neutrophil composition, with the proportion of Thbs1^hi^ neutrophils increasing notably at 7 days post-ESWT and Gbp2^hi^ neutrophils becoming enriched at 14 days post-ESWT. Specifically, at ESWT day 7, Thbs1^hi^ neutrophils accounted for 66.2% of the population, accompanied by a reduction in S100a8^hi cells, suggesting a transition toward a reparative phenotype. By ESWT day 14, Gbp2^hi^ neutrophils increased to 66.4%, while S100a8^hi^ cells were further reduced, indicating resolution of inflammation and potential engagement of interferon-related immune regulation. These temporal shifts implied that ESWT accelerated the inflammatory-to-reparative transition of neutrophils in DWs, characterized by an early enrichment of Thbs1^hi^ pro-angiogenic neutrophils and a later dominance of Gbp2^hi^ interferon-responsive neutrophils, which might collectively contribute to improved vascularization and wound closure.

GO enrichment analysis revealed distinct functional specializations among the three neutrophil subsets (Figure 6D). S100a8^hi^ neutrophils were predominantly enriched in chemotaxis-related biological processes, including leukocyte chemotaxis, granulocyte chemotaxis, and regulation of neutrophil chemotaxis, reflecting their strong pro-inflammatory and immune cell recruitment capacity. In contrast, Thbs1^hi^ neutrophils showed enrichment in metabolic and stress-response pathways, such as NADH regeneration, ADP catabolic process, and cellular response to hypoxia, suggesting their involvement in tissue repair and adaptation to the wound microenvironment. Gbp2^hi^ neutrophils were significantly associated with ribosome-related biogenesis processes (ribosomal small subunit biogenesis, ribonucleoprotein complex biogenesis, ribosome biogenesis), indicating enhanced protein synthesis potential, which may support interferon-mediated immune regulation during the late stages of healing. AUCell analysis based on NETosis-related gene sets revealed distinct activity patterns among neutrophil subtypes (Figure 6E). S100a8^hi^ neutrophils exhibited the highest NETosis scores, followed by Thbs1^hi^ and Gbp2^hi^ neutrophils, indicating a predominant pro-NETosis phenotype in the inflammatory S100a8^hi^ population. When stratified by experimental groups, NETosis scores were elevated in the DW group compared to controls, suggesting enhanced NET formation potential in chronic wounds. ESWT treatment, particularly at day 14, was associated with a reduction in NETosis scores, indicating that ESWT might attenuate excessive NETosis activity during the healing process. These results suggest that ESWT not only reshaped neutrophil subtype composition but also modulated their NETosis potential, potentially contributing to inflammation resolution in diabetic wounds.

To identify the molecular pathways through which ESWT modulated neutrophil biology during DW healing, we performed differential expression and functional enrichment analyses comparing control and ESWT-treated groups with untreated DW groups. Venn analysis of DEGs revealed both shared and unique transcriptional responses across comparisons (Figure 6F). Specifically, 283 DEGs were common to all three comparisons (ESWT 7d vs. DW, ESWT 14d vs. DW, and Control vs. DW), while 110 and 105 DEGs were unique to ESWT 7d and ESWT 14d, respectively, indicating stage-specific molecular signatures induced by ESWT.

As shown in Figure 6G, the enrichment of regulation of endocytosis suggested that neutrophils were actively involved in the internalization of pathogens, debris, and apoptotic cells at the wound site. This was crucial for both the clearance of infection and the resolution of inflammation during the wound healing process. Endocytosis also played a role in antigen presentation, which was important for initiating adaptive immunity. Neutrophil migration and neutrophil chemotaxis were closely related processes, both critical for neutrophils’ ability to migrate toward the wound site in response to inflammatory signals. Chemotaxis was primarily driven by cytokines and other signaling molecules, such as IL-8 and C5a, which guided neutrophils to the site of injury or infection. The enrichment of these processes suggested that neutrophils in the DW environment were highly responsive to signaling cues, which was important for a rapid immune response and infection control. The structural constituent of cytoskeleton process highlighted the importance of the cytoskeleton in neutrophil movement and shape changes. Neutrophils relied on actin polymerization and other cytoskeletal dynamics to change shape, migrate, and interact with other cells and the ECM. This process was vital for neutrophil motility, enabling them to effectively navigate through tissue barriers and reach the site of infection or injury. KEGG pathway enrichment (Figure 6H) highlighted regulation of actin cytoskeleton, endocytosis, chemokine signaling pathway, and Fc gamma R-mediated phagocytosis as top pathways, alongside infection-related pathways (*Salmonella* infection, *Yersinia* infection), indicating that ESWT might enhance neutrophil migratory capacity, phagocytic activity, and immune defense mechanisms. Collectively, these results suggested that ESWT induced both common and time-specific transcriptional programs in neutrophils, characterized by cytoskeletal remodeling, enhanced chemotaxis, and pathogen clearance, which might accelerate the inflammatory-to-repair transition in DW healing.

**FIGURE 7:**
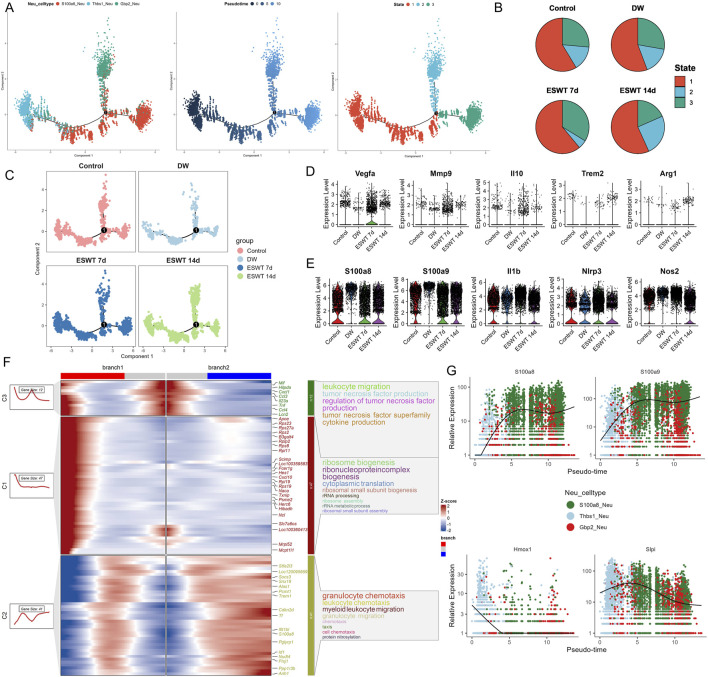
ESWT promoted neutrophil phenotypic transition and alleviated pro-inflammatory activation during DW healing. **(A)** Pseudotime trajectory analysis of neutrophil subclusters. Cells were ordered along pseudotime (middle) and classified into distinct states (right), indicating dynamic transitions of neutrophil phenotypes. **(B)** Pie charts showing pseudotime state distributions in control, DW, ESWT 7d, and ESWT 14d groups, highlighting that ESWT shifted neutrophils toward later states associated with tissue repair. **(C)** Pseudotime distribution plots of neutrophils across groups, demonstrating that ESWT remodeled trajectories compared with DW. **(D)** Violin plots of representative reparative and anti-inflammatory genes (Vegfa, Mmp9, Il10, Trem2, Arg1), showing increased expression after ESWT treatment. **(E)** Violin plots of pro-inflammatory and stress-related genes (S100a8, S100a9, Il1b, Nlrp3, Nos2), which were reduced by ESWT, indicating suppression of excessive inflammatory activation. **(F)** Branched heatmap of pseudotime-dependent gene modules. Cluster 1 genes were enriched in ribosome biogenesis and protein translation, cluster 2 genes in granulocyte chemotaxis and cluster 3 genes in leukocyte migration and TNF signaling, reflecting ESWT-driven reprogramming of neutrophil function. **(G)** Smoothed pseudotime expression trends of representative genes (S100a8, S100a9, Hmox1, Slpi), showing that ESWT downregulated inflammatory mediators while upregulating protective and reparative genes.

To explore the potential differentiation continuum among neutrophil subtypes during DW healing, we performed pseudotime trajectory analysis (Figure 7A). The reconstructed trajectory revealed a continuous progression from pro-inflammatory S100a8^hi^ neutrophils through angiogenesis-associated Thbs1^hi^ neutrophils to interferon-responsive Gbp2^hi^ neutrophils, suggesting a sequential phenotypic transition.

Pseudotime analysis revealed that Gbp2^hi^ neutrophils were enriched at the earliest stage (state 1), preceding the predominance of S100a8^hi^ neutrophils at intermediate and late stages, while Thbs1^hi^ neutrophils emerged primarily at the terminal stage, suggesting a sequential shift in neutrophil phenotypes during wound healing. To investigate the impact of ESWT on neutrophil state dynamics during DW healing, pseudotime trajectories were reconstructed and colored by experimental group (Figure 7B). In the control group, neutrophils were predominantly distributed in state 1, with smaller proportions in states 2 and 3. In DW, there was a marked increase in state 3 cells, accompanied by a reduction in state 1 cells, suggesting a shift toward terminal-stage phenotypes in the absence of ESWT. Following ESWT treatment, the distribution of neutrophil states was altered. At ESWT day 7, state 1 cells became dominant, while states 2 and 3 were substantially reduced, indicating an early-stage bias in the trajectory. By ESWT day 14, although state 1 cells remained prevalent, there was a modest recovery of states 2 and 3, suggesting partial progression along the trajectory (Figure 7C). Analysis of key neutrophil-related repair genes showed that Vegfa, Mmp9, Il10, Trem2, and Arg1 maintained comparable expression levels across groups, with a slight increase after ESWT treatment (Figure 7D). Pro-inflammatory markers S100a8, S100a9, Il1b, Nlrp3, and Nos2 were highly expressed in the DW group, while ESWT treatment (especially at day 14) tended to reduce their expression, indicating a potential alleviation of inflammatory activation (Figure 7E).

Branch analysis of the pseudotime trajectory identified three major gene clusters with distinct temporal patterns along the two main branches (Figure 7F). Cluster C3 genes were highly expressed in branch 1 and enriched in immune activation processes, including leukocyte migration, tumor necrosis factor production, and cytokine production, indicating a strong pro-inflammatory profile. Cluster C1 genes were enriched in branch 1 and predominantly associated with ribosome biogenesis, ribonucleoprotein complex biogenesis, and cytoplasmic translation, suggesting elevated protein synthesis capacity. Cluster C2 genes were enriched in branch 2, with GO terms related to granulocyte chemotaxis, myeloid leukocyte migration, and protein nitrosylation, pointing to a role in immune cell trafficking and tissue adaptation. Gene expression dynamics along pseudotime revealed that S100a8 and S100a9 were progressively upregulated toward the later stages, consistent with their association with pro-inflammatory neutrophils. In contrast, HmoX1 showed early-stage enrichment, suggesting a stress-response role, while Slpi peaked at intermediate stages before declining, indicating potential involvement in the resolution phase (Figure 7G). These results suggested that ESWT reshaped the temporal distribution of neutrophil phenotypes, potentially promoting a coordinated transition along the inflammatory–reparative continuum, with an initial enrichment of early-stage cells followed by gradual re-entry into intermediate and late states.

### Keratinocyte reprogramming underlied ESWT-mediated epidermal repair in DWs

Single-cell transcriptomic profiling identified six distinct KCs subpopulations, including Krtdap^hi^, Igfbp2^hi^, Nos2^hi^, Tgm5^hi^, Mt4^hi^, and Gzmk^hi^ (Supplementary Figure S3A). Among these, Krtdap^hi^ represented the predominant subset, followed by Igfbp2^hi^ and Nos2^hi^, while Gzmk^hi^ accounted for the smallest fraction (Supplementary Figure S3B). In the DW group, the proportions of most KC subsets markedly decreased, indicating impaired keratinocyte function in DWs. Following ESWT intervention, especially at day 14, multiple subsets exhibited a pronounced recovery in proportion. Notably, Krtdap^hi^ and Tgm5^hi^ were substantially enriched in the ESWT 14d group, while inflammation-related Nos2^hi^ and stress-responsive Mt4^hi^ also showed partial restoration (Supplementary Figure S3C). These findings suggested that ESWT reshaped the KC landscape in DW, facilitating barrier repair and functional recovery to promote wound healing.

Functional enrichment analysis of KC subpopulations revealed distinct biological roles associated with each cluster (Supplementary Figure S3D). Krtdap^hi^ was enriched in skin barrier establishment, epidermis development, and gland morphogenesis, while Igfbp2^hi^ was associated with T cell differentiation and lymphocyte activation. Nos2^hi^ and Tgm5^hi^ were enriched in cytokine-mediated signaling, leukocyte activation, and chemotaxis, reflecting their potential roles in immune modulation. Mt4^hi^ showed enrichment in cellular responses to biotic stimuli, and Gzmk^hi^ was linked to immune activation and cytotoxicity-related pathways. AUCell scoring based on inflammation-related markers revealed that KCs in the DW group exhibited significantly elevated inflammatory activity compared to controls (Supplementary Figure S3E). ESWT treatment markedly reduced the inflammation score, with the most pronounced suppression observed at 14 days. Conversely, AUCell scoring of differentiation-associated markers showed that differentiation activity was reduced in the DW group relative to controls (Supplementary Figure S3F). ESWT intervention, particularly at 7 days, partially restored differentiation potential, although scores at 14 days remained lower than controls. These findings indicated that ESWT attenuated excessive inflammatory responses while promoting the reactivation of KC differentiation programs, thereby contributing to epidermal repair in DWs.

Comparative differential expression analysis (Supplementary Figure S3G) identified 161 common genes altered in control vs. DW, ESWT 7d vs. DW and ESWT 14d vs. DW, suggesting shared ESWT-responsive mechanisms.

As shown in Supplementary Figure S3H, the enrichment of the cellular response to molecules of bacterial origin suggested that KCs played an active role in recognizing and responding to pathogen-associated molecular patterns (PAMPs) in the wound environment. This response is critical for initiating the innate immune response and activating inflammatory pathways that promote tissue repair and defense against infection. Regulation of cell-cell adhesion is a key process for KCs, particularly in the context of wound healing, where KCs need to migrate and proliferate to cover the wound site. The enrichment in this process indicated that KCs were involved in modulating adhesion molecules, such as cadherins and integrins, to facilitate migration, epithelialization, and wound closure. The humoral immune response enrichment pointed to the involvement of KCs in immune signaling, likely through the release of cytokines and chemokines that influence the recruitment and activation of immune cells, such as T cells and macrophages. This supports the idea that KCs are active players in the local immune response during wound healing, contributing to both innate and adaptive immunity. The regulation of T cell activation was another enriched process, suggesting that KCs may interact with immune cells, particularly T cells, in the wound bed. This interaction is essential for coordinating the immune response and ensuring proper wound healing. KCs, through the secretion of cytokines and other factors, could influence T cell activation and function, which may contribute to tissue remodeling and the resolution of inflammation.

The KEGG analysis highlighted the regulation of cell-cell adhesion as a key pathway, reinforcing the idea that KCs were involved in maintaining the integrity of the epithelial barrier and facilitating interactions with neighboring cells (Supplementary Figure S3I). The regulation of these adhesion molecules is crucial for both wound closure and immune cell communication within the wound microenvironment. The NOD-like receptor (NLR) signaling pathway was also enriched, suggesting that KCs might participate in the recognition of intracellular pathogens or stress signals. NLRs are involved in detecting damage-associated molecular patterns (DAMPs) and activating inflammatory responses, which are crucial for managing infection and promoting tissue repair. Finally, the IL-17 signaling pathway was enriched, indicating the involvement of keratinocytes in the inflammatory process through the production of IL-17 and other cytokines. This pathway is important for mediating inflammation and recruiting neutrophils to the wound site, and it plays a significant role in the defense against infection and the regulation of tissue repair. These findings provide valuable insights into the complex role of KCs in wound healing, particularly in the context of chronic wounds or diabetic ulcers, where immune dysregulation can impede healing.

Pseudotime trajectory analysis delineated a continuous differentiation path of KCs, revealing distinct branch points and state transitions (Supplementary Figure S4A). The distribution of KC subtypes along the trajectory showed that Krtdap^hi^, Igfbp2^hi^, and Nos2^hi^ primarily enriched the intermediate branches, whereas Tgm5^hi^, Mt4^hi^, and Gzmk^hi^ occupied in terminal states (Supplementary Figure S4B). State composition analysis demonstrated that the DW group was enriched in late-stage states, indicative of altered or delayed differentiation, while ESWT treatment, particularly at 14 days, shifted the distribution toward early and intermediate states, suggesting accelerated KC differentiation and functional recovery (Supplementary Figure S4C). These results indicated that ESWT promoted a reprogramming of KC trajectories, potentially restoring balanced epidermal renewal and barrier function during DW healing. Differential expression analysis revealed that inflammation-related genes, including Nos2, Il1b, Cxcl2, S100a8, S100a9, and Ptgs2, were markedly upregulated in DW keratinocytes compared to controls, while epidermal differentiation markers such as Krt1, Krt10, Sprr1a, Evpl, Ppl, and Dsg1 were downregulated (Supplementary Figures S4D,E). ESWT treatment, particularly at 14 days, significantly reduced the expression of pro-inflammatory genes and restored the expression of differentiation-associated genes, suggesting a shift toward a more reparative keratinocyte phenotype. Branched heatmap analysis along pseudotime trajectories (Supplementary Figure S4F) identified distinct gene modules linked to inflammatory responses and epidermal development. Gene Ontology enrichment highlighted functional transitions from immune activation to structural repair during ESWT-mediated healing. Pseudotime expression dynamics further demonstrated that Krt1 and Krt10 expression increased toward the later stages of differentiation, whereas inflammatory mediators S100a8 and S100a9 were enriched at earlier pseudotime states (Supplementary Figure S4G). These results indicated that ESWT promoted a coordinated suppression of inflammatory programs and activation of epidermal differentiation in KCs, facilitating barrier restoration in DWs.

### ESWT-induced functional diversity and angiogenesis potential of EC subclusters

**FIGURE 8:**
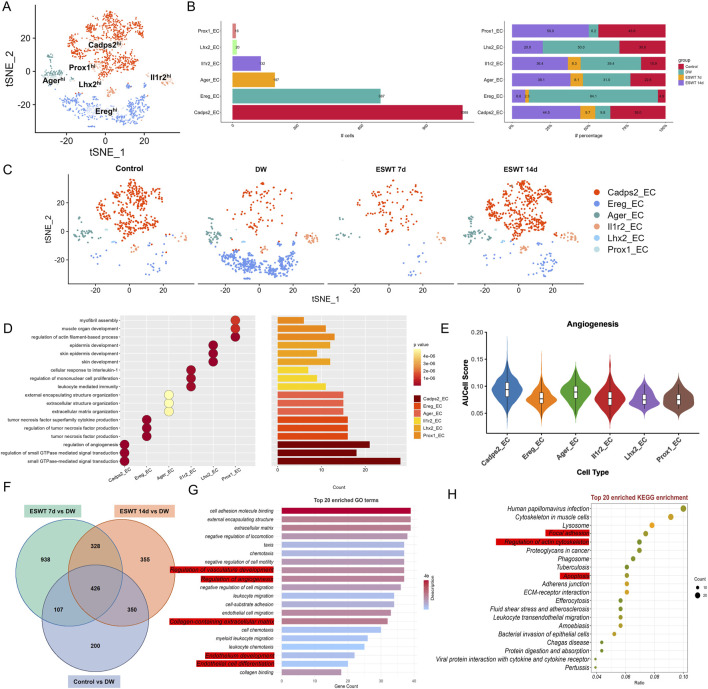
ESWT reprogrammed EC subclusters and enhanced angiogenic activity in diabetic wounds. **(A)** t-SNE plot showing six EC subclusters defined by marker gene expression. **(B)** Bar plots showing the absolute (left) and relative (right) proportions of EC subclusters across control, DW, ESWT 7d, and ESWT 14d groups, indicating that ESWT restored endothelial heterogeneity and promoted pro-angiogenic subclusters. **(C)** t-SNE visualization of EC distribution in each group, showing marked loss of endothelial subsets in DW and progressive restoration following ESWT treatment. **(D)** Functional enrichment analysis of EC subclusters, with GO terms enriched in myofibril assembly, endothelial cell migration, angiogenesis, and extracellular matrix organization. **(E)** Violin plots of AUCell scores for angiogenesis-related gene signatures across EC subclusters, showing that ESWT increased angiogenic potential, especially in Cadps2_EC and Ager_EC. **(F)** Venn diagram of DEGs between control, DW, ESWT 7d, and ESWT 14d groups, showing substantial overlap and ESWT-specific transcriptional changes. **(G)** Top 20 enriched GO terms of DEGs, including extracellular matrix organization, endothelial cell differentiation, and regulation of angiogenesis. **(H)** Top 20 enriched KEGG pathways of DEGs, such as focal adhesion, ECM-receptor interaction, PI3K-Akt signaling, leukocyte transendothelial migration, and fluid shear stress, indicating that ESWT activated key endothelial pathways to promote vascular regeneration.

Single-cell transcriptomic profiling identified 6 EC subclusters in wound tissues, each characterized by distinct transcriptional signatures and biological functions. As shown in Figure 8A, unbiased clustering of ECs across all samples resolved six transcriptionally distinct subclusters. Group composition analysis revealed that DW samples predominated in Ereg^hi^ (84.1%) and Il1r2^hi^ ECs, whereas these proportions markedly decreased after ESWT treatment (Figure 8B). In contrast, ESWT 14d contributed the largest fraction of Prox1^hi^ EC (50.0%) compared with only 6.2% in DW. Il1r2^hi^ ECs showed high proportions in DW (39.4%) and ESWT 14d (36.4%) but lower in control (15.9%) and ESWT 7d (8.3%), consistent with an anti-inflammatory IL-1 signaling profile. Lhx2^hi^ ECs were enriched in DW (50.0%) and decreased after ESWT 14d (20.0%), while nearly absent in ESWT 7d. Ager^hi^ ECs were most abundant in ESWT 14d (38.1%) and DW (31.0%), with lower representation in control (22.8%) and ESWT 7d (8.1%). Collectively, ESWT shifted the EC landscape from DW-associated Ereg^hi^, Lhx2^hi^ and partially Il1r2^hi^ ECs toward pro-angiogenic phenotype (Cadps2_EC) and lymphangiogenic (Prox1_EC) states, indicating that ESWT promoted vascular remodeling while alleviating inflammation, thereby accelerating DW healing (Figure 8C).

GO enrichment analysis of the top three biological processes for each EC cluster revealed distinct functional specializations (Figure 8D). Cadps2^hi^ ECs were strongly associated with “regulation of angiogenesis,” “regulation of small GTPase mediated signal transduction,” and “small GTPase-mediated signal transduction,” consistent with active vascular remodeling and cytoskeletal regulation. Ereg^hi^ ECs were enriched in immune and inflammatory-related processes including “tumor necrosis factor superfamily cytokine production” and its regulation, suggesting a pro-inflammatory or immune-modulatory role. Ager^hi^ ECs showed enrichment in extracellular structure organization terms, such as “extracellular matrix organization” and “external encapsulating structure organization,” indicating potential involvement in matrix remodeling. Il1r2^hi^ ECs were associated with “cellular response to interleukin-1,” “regulation of mononuclear cell proliferation,” and “leukocyte mediated immunity,” highlighting an immune-regulatory phenotype. Lhx2^hi^ ECs were linked to skin-related processes, including “epidermis development” and “skin development,” while Prox1^hi^ ECs were enriched for “myofibril assembly” and “muscle organ development,” suggestive of roles in vascular smooth muscle differentiation. Notably, AUCell analysis for the angiogenesis gene set showed that Cadps2^hi^ ECs had the highest angiogenic activity, followed by Ager^hi^ and Il1r2^hi^ ECs, whereas Ereg^hi^, Lhx2^hi^, and Prox1^hi^ ECs displayed relatively lower angiogenesis scores (Figure 8E), supporting the functional heterogeneity of EC subtypes in ESWT-mediated wound healing.

To identify common EC programs enhanced by ESWT, we compared each group with the DW group and extracted the intersection of upregulated genes across all comparisons (ESWT 7d vs. DW, ESWT 14d vs. DW, and Control vs. DW). Venn analysis revealed 426 shared upregulated genes among the three comparisons (Figure 8F). As shown in Figure 8G, the enrichment of regulation of vasculature development and regulation of angiogenesis highlighted the fundamental role ECs play in the formation of new blood vessels. Angiogenesis is a key process in wound healing, ensuring an adequate supply of oxygen and nutrients to the regenerating tissue. The regulation of vasculature development further emphasized the involvement of ECs in maintaining and enhancing the vascular network during tissue repair. Endothelial cell differentiation and endothelium development are closely related processes that point to the ability of ECs to adapt and change during wound healing. These processes enabled ECs to form new vessels, undergo proliferation, and maintain the structure of the endothelial lining of blood vessels. The enriched expression of these processes suggested that ECs in the wound environment actively participate in the regeneration and stabilization of the blood vessel network. The enrichment of collagen-containing extracellular matrix indicated that ECs contributed to ECM remodeling, an essential step for the development of new blood vessels and tissue regeneration. Collagen, a key ECM protein, plays a significant role in providing structural support to blood vessels, which is crucial for their stability and function. This suggested that ECs not only participate in angiogenesis but also help in organizing and remodeling the ECM, facilitating efficient wound closure and tissue repair. KEGG pathway analysis further highlighted enrichment of cytoskeletal organization and adhesion pathways, vesicular trafficking and turnover, and immune processes (Figure 8H). These functional programs collectively suggested that ESWT activated a conserved pro-repair network in ECs, coordinating cytoskeletal remodeling, matrix interactions, vascular regeneration, and inflammation resolution, thereby accelerating DW healing.

**FIGURE 9:**
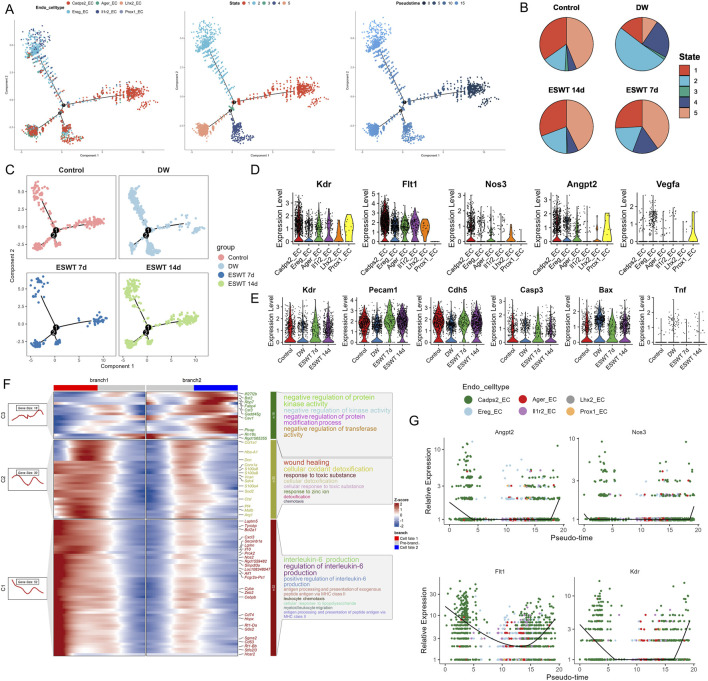
ESWT promoted EC phenotypic transition and activated pro-angiogenic programs in DWs. **(A)** Pseudotime trajectory analysis of EC subclusters. Cells were ordered along pseudotime (middle) and grouped into distinct states (right), revealing dynamic endothelial transitions. **(B)** Pie charts showing pseudotime state distributions in control, DW, ESWT 7d, and ESWT 14d groups, indicating ESWT-induced remodeling of EC states toward reparative phenotypes. **(C)** Pseudotime distribution plots for each group, demonstrating that ECs in ESWT groups progressed into later states associated with angiogenesis and vascular repair. **(D)** Violin plots of pro-angiogenic genes (Kdr, Flt1, Nos3, Angpt2, Vegfa), showing upregulation following ESWT treatment. **(E)** Violin plots of vascular function- and apoptosis-related genes (Kdr, Pecam1, Cdh5, Casp3, Bax, Tnf), revealing enhanced endothelial activity and reduced apoptosis under ESWT. **(F)** Branched heatmap of pseudotime-dependent gene modules. Cluster 1 genes were enriched in IL-6 production and inflammatory responses, branch 2 in wound healing and detoxification, and branch 3 in kinase activity and protein regulation, indicating functional divergence during EC reprogramming. **(G)** Smoothed pseudotime expression of representative angiogenic genes (Angpt2, Nos3, Flt1, Kdr) across EC subclusters, showing progressive activation of pro-angiogenic signatures under ESWT.

Pseudotime trajectory analysis of ECs using Monocle2 revealed a branched differentiation pattern comprising five distinct states (Figure 9A). Mapping of EC subtypes onto the trajectory showed that Cadps2^hi^ ECs occupied early pseudotime positions, while other clusters were distributed along intermediate branches, and Il1r2^hi^ ECs were enriched in later pseudotime regions. State-specific mapping indicated that State 2 was predominantly composed of Ereg_EC, State 1 was enriched in Cadps2^hi^ ECs, and State 5 contained higher proportions of Cadps2^hi^ and Ager^hi^ ECs, suggesting functional divergence along the trajectory. Comparison of state distributions across experimental groups showed that DW samples were dominated by State 2 with reduced representation of State 1, whereas ESWT treatment shifted the composition toward State 1 and State 5 (Figure 9B). ESWT 14d samples exhibited a balanced distribution across pro-angiogenic states, while ESWT 7d showed a partial transition from the DW state profile. These results indicated that ESWT promoted a pseudotime transition of ECs from a DW-associated Ereg_EC-dominant state toward pro-angiogenic phenotypes, potentially facilitating vascular remodeling during wound healing. Monocle2 trajectory analysis revealed two major differentiation branches among EC subpopulations in DW tissues (Figure 9C). In the Control group, ECs were relatively balanced between Branch 1 and Branch 2, whereas the DW group displayed a marked shift toward Branch 2. ESWT treatment altered this trajectory pattern, with ESWT 7d and ESWT 14d groups showing increased cell distribution in Branch 1, suggesting a transition toward a pro-repair developmental path.

Violin plot analysis demonstrated heterogeneous expression patterns of angiogenesis- and endothelial function–related genes among EC subclusters (Figure 9D). Prox1_EC exhibited the highest expression of Vegfa and Angpt2, while Lhx2_EC and Il1r2_EC showed elevated Flt1 and Kdr levels. Across treatment groups, ESWT significantly enhanced the expression of vascular integrity markers Pecam1 and Cdh5, as well as angiogenesis-related genes Kdr and Flt1, compared with the DW group (Figure 9E). Conversely, pro-apoptotic genes Casp3 and Bax, along with the pro-inflammatory cytokine Tnf, were markedly elevated in the DW group but reduced after ESWT, with ESWT 14d showing the lowest levels. BEAM analysis identified three major gene clusters (C1–C3) with distinct expression patterns along the two endothelial cell branches (Figure 9F). Cluster C1 genes were highly expressed in Branch 1 and included multiple pro-angiogenic and endothelial activation markers (Cxcl3, Pf4, Mafb, Arg1). GO enrichment indicated strong associations with wound healing, cellular oxidant detoxification, and chemotaxis, suggesting a reparative and pro-migratory phenotype. Cluster C2 genes were upregulated in Branch 2, enriched for interleukin-6 production, antigen presentation, and leukocyte chemotaxis, indicating a more inflammatory and immune-interacting state. Cluster C3 contained genes showing moderate changes between branches, related to negative regulation of protein kinase activity and response to zinc ion. Pseudotime analysis revealed that angiogenesis-related genes (Angpt2, Nos3, Flt1, Kdr) displayed a clear upregulation trend along the Branch 1 trajectory, particularly toward the late pseudotime stages, while remaining at lower levels in Branch 2 (Figure 9G). This pattern suggested that Branch 1 represented a pro-angiogenic and tissue-repairing endothelial state, whereas Branch 2 was skewed toward inflammation and immune response. Collectively, these findings indicated that ESWT might promote ECs to preferentially adopt the Branch 1 trajectory, reprogramed EC subpopulations toward an angiogenesis-promoting, survival-favoring phenotype while suppressing apoptosis and inflammation, thereby contributing to accelerated DW healing.

### ESWT reshaped SMC heterogeneity and promoted a contractile reparative phenotype in DW healing

To explore the effects of ESWT on SMC heterogeneity in DWs, we performed tSNE clustering, identifying six distinct SMC subtypes: Sncg^hi^, Trem1^hi^, Postn^hi^, Top2a^hi^, Nkg7^hi^, and Bcl11a^hi^ (Supplementary Figure S5A). Sncg^hi^ constituted the largest proportion of cells, followed by Trem1^hi^ and Postn^hi^. In DW tissues, there was an increased proportion of Trem1^hi^ and a marked reduction in Sncg^hi^ compared with the control (Supplementary Figure S5B). ESWT treatment notably reshaped the SMC landscape: ESWT 7d decreased the proportion of Trem1^hi^, while ESWT 14d further expanded Sncg^hi^ and Postn^hi^, both of which are linked to extracellular matrix organization and vessel stabilization (Supplementary Figure S5C). These findings suggested that ESWT promoted DW healing, at least in part, by selectively enhancing SMC subpopulations that contribute to vascular repair and tissue remodeling.

To further clarify the biological functions of SMC subtypes in the context of DW healing, we carried out GO enrichment analysis based on the top gene sets in each subclusters (Supplementary Figure S5D). GO term analysis indicated that Sncg^hi^ were enriched in muscle relaxation and glycoprotein biosynthetic processes, Trem1^hi^ in granulocyte and leukocyte chemotaxis, and Postn^hi^ in extracellular matrix and structural organization. Top2a^hi^ were strongly associated with cell cycle–related processes, including chromosome segregation and proliferation of lymphocytes, mononuclear cells, and leukocytes. Nkg7^hi^ and Bcl11a^hi^ were primarily enriched in immune-related terms, such as regulation of B cell activation and lymphocyte proliferation. AUCell scoring revealed a marked reduction in the contraction phenotype score of SMCs in DW compared with controls, indicating loss of contractile function. ESWT treatment restored contraction scores in a time-dependent manner, with the most pronounced recovery observed at 14 days, approaching control levels (Supplementary Figure S5E). In contrast, the inflammation phenotype score was significantly elevated in DW, reflecting heightened inflammatory activation of SMCs. ESWT progressively reduced inflammation scores, with ESWT 14d showing the lowest values among all groups (Supplementary Figure S5F). These results demonstrated that ESWT promoted phenotypic remodeling of SMCs from an inflammation-dominated state toward a contractile, vascular-supportive phenotype, potentially contributing to improved vascular function and DW repair.

Venn diagram analysis identified 85 overlapping differentially expressed genes among the control vs. DW, ESWT 7d vs. DW, and ESWT 14d vs. DW comparisons, highlighting a core gene set modulated by ESWT (Supplementary Figure S5G).

As shown in Supplementary Figure S5H, the enrichment of the chemokine-mediated signaling pathway and leukocyte chemotaxis suggested that SMCs were involved in orchestrating the migration of immune cells to the wound site. Chemokines are signaling molecules that recruit immune cells, such as neutrophils and macrophages, to the site of injury. This is particularly important for inflammation resolution and initiating the repair process. The presence of humoral immune response further indicated that SMCs might be actively involved in immune cell activation and cytokine production, modulating the inflammatory response in the wound microenvironment. The enrichment of the response to toxic substance process suggested that SMCs might also play a role in responding to oxidative stress or other harmful substances generated during injury. These responses are crucial for protecting the tissue from further damage and initiating tissue repair mechanisms. The antigen processing and presentation pathway enrichment indicated that SMCs might contribute to antigen presentation, a key process in adaptive immunity (Supplementary Figure S5I). By presenting antigens to T cells, SMCs could facilitate the activation of immune responses that are necessary for wound healing and infection control. The enrichment of the IL-17 signaling pathway suggested that SMCs were involved in inflammatory signaling through the production of IL-17 and other cytokines. IL-17 is a potent mediator of inflammation and plays a role in recruiting neutrophils to the wound site, enhancing tissue defense mechanisms. The NF-κB signaling pathway is critical for regulating inflammation and immune responses. Its enrichment suggested that SMCs might influence the inflammatory process through the activation of NF-κB, which regulated the expression of pro-inflammatory cytokines and chemokines. The cytokine-cytokine receptor interaction pathway was involved in the signaling between immune cells and surrounding tissue, coordinating the inflammatory response and tissue repair. This pathway indicated that SMCs were likely involved in cell-cell communication within the wound environment, helping to coordinate immune cell recruitment and tissue remodeling. Finally, the enrichment of apoptosis suggested that SMCs were involved in the regulation of cell death processes, which is important for the resolution of inflammation and the removal of damaged or dysfunctional cells during wound healing. These results suggested that ESWT promoted DW healing by modulating SMC subsets, which played key roles in immune cell recruitment, regulation of inflammatory responses, tissue remodeling, and extracellular matrix reorganization—critical processes that collectively contribute to efficient wound healing.

Pseudotime trajectory analysis demonstrated a branched developmental continuum of SMCs (Supplementary Figure S6A). Sncg^hi^ SMCs were primarily located at the early and intermediate branches, whereas Postn^hi^ SMCs and Trem1^hi^ SMCs occupied late terminal regions, suggesting distinct roles in tissue remodeling and vascular function. State mapping revealed that DW tissues exhibited a higher proportion of cells in early states (State 1 and State 3) compared with controls. ESWT treatment shifted the SMC distribution toward intermediate and late reparative states, particularly at ESWT 14d, where a marked increase in States 6 and 8 was observed (Supplementary Figure S6B). Group-specific trajectory plots confirmed that ESWT promoted a transition from pro-inflammatory to reparative phenotypes along the pseudotime axis, indicating its role in facilitating SMC maturation and functional specialization DW healing (Supplementary Figure S6C). Violin plot analysis revealed distinct expression changes in inflammatory and contractile markers of SMCs across groups. Inflammatory genes, including Il6, Cxcl2, Tnf, Nfkb1, and Ptgs2, were markedly upregulated in DW compared with controls, indicating an activated pro-inflammatory state. ESWT treatment, particularly at 14 days, substantially reduced the expression of these inflammatory mediators, suggesting suppression of excessive inflammation (Supplementary Figure S6D). Conversely, contractile markers Acta2, Myh11, Tagln, Cnn1, and Des were downregulated in DW, reflecting loss of SMC contractile phenotype during chronic wound pathology. ESWT, especially at 14 days, restored the expression of these contractile genes toward control levels, indicating a phenotypic shift back to a vascular-supportive and structurally stable state (Supplementary Figure S6E). These findings suggested that ESWT promoted DW healing by simultaneously attenuating inflammatory activation and enhancing the contractile phenotype of SMCs, thereby supporting vascular integrity and tissue repair.

BEAM analysis at node 2 revealed that ESWT modulated the bifurcation of SMCs into two distinct functional trajectories (Supplementary Figure S6F). In the immune-oriented branch (cell fate 1), genes enriched in granulocyte chemotaxis and migration (Cxcl2, S100a8, S100a9) were highly expressed in DW, indicating a pro-inflammatory state. ESWT treatment, particularly at 14 days, reduced the dominance of this branch, suggesting suppression of excessive inflammatory recruitment. In contrast, the reparative branch (cell fate 2) showed upregulation of genes related to connective tissue development, actomyosin structure organization, and calcium ion transmembrane transport via high voltage-gated calcium channels (Actb, Myl9, Tpm2, Vim, Cacna1c, Plcl1). ESWT enhanced the proportion of SMCs adopting this trajectory, promoting structural remodeling, contractile function, and vascular stabilization. Pseudotime expression analysis revealed that contractile markers Acta2 and Myh11 decreased along the trajectory, whereas inflammatory and chemotactic genes such as Cxcl2 and Nfkb1 were upregulated toward reparative and immune-modulatory states (Supplementary Figure S6G). These findings indicate that ESWT facilitated a phenotypic shift at node 2 from a pro-inflammatory state toward a repair-oriented, contractile, and vascular-supportive SMC phenotype, thereby contributing to improved DW healing.

### ESWT modulated the distribution and composition of immune cells

**FIGURE 10:**
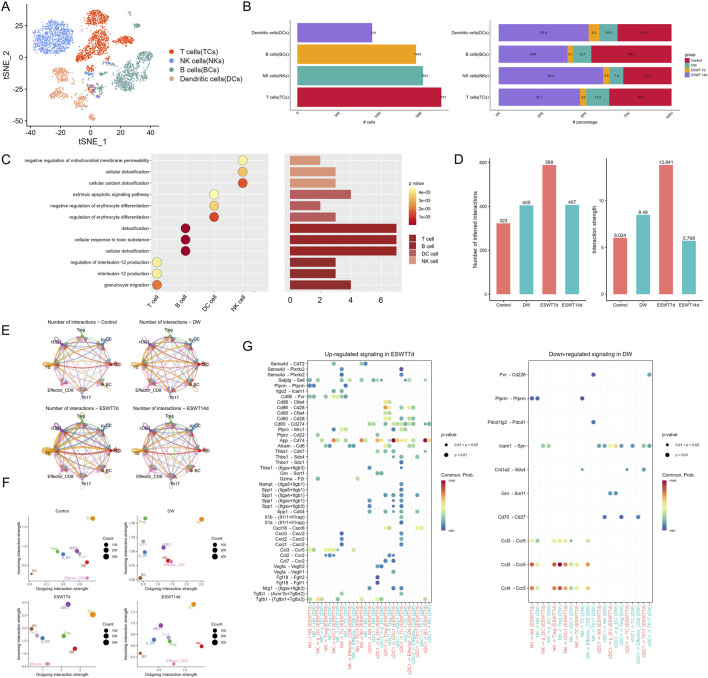
ESWT modulated immune cell composition and enhanced intercellular communication in DWs. **(A)** t-SNE plot showing four immune cell subtypes: TCs, NKs, BCs, and DCs. **(B)** Bar plots of absolute (left) and relative (right) numbers of each immune cell type across control, DW, ESWT 7d, and ESWT 14d groups, showing dynamic remodeling of immune cells under ESWT. **(C)** Functional enrichment analysis of immune cell subsets, highlighting pathways related to cell activation, differentiation, immune regulation, and chemotaxis. **(D)** Quantification of total and significant ligand–receptor interactions among immune cells, showing reduced interactions in DW and partial restoration by ESWT treatment. **(E)** Circle plots of cell–cell communication networks in each group, with edge thickness indicating interaction strength. ESWT groups showed enhanced connectivity compared with DW. **(F)** Scatter plots of incoming and outgoing interaction strength across immune cell types in control, DW, ESWT 7d, and ESWT 14d groups, indicating that ESWT increased immune cell communication activity. **(G)** Bubble plots of upregulated signaling pathways in ESWT 7d (left) and downregulated signaling pathways in DW (right), showing that ESWT enhanced key immune regulatory signals while DW was associated with reduced immune cell signaling.

To characterize the immune landscape in DWs and its response to ESWT, we focused on TCs, BCs, NKs, and DCs. Although DCs were derived from the myeloid lineage, they were analyzed together with lymphoid immune cells in this study due to their central role in immune activation and communication within the wound microenvironment. Unsupervised t-SNE projection revealed clear segregation of these immune populations (Figure 10A). Quantitative analysis showed that TCs accounted for the largest fraction, followed by NKs, BCs, and DCs (Figure 10B). Further comparison among experimental groups demonstrated that the relative abundance of each immune cell type was dynamically remodeled after ESWT treatment. Specifically, DCs dominated in the Control group (31.2%), whereas their proportion markedly decreased in DW. ESWT administration gradually restored the balance, with a notable increase in all immune cells at day 14. These findings suggested that ESWT promoted a shift in the immune microenvironment, enhancing lymphocyte and antigen-presenting cell representation to facilitate wound repair. As shown in Figure 10C, the analysis of biological process enrichment highlighted the pivotal role of immune cells, particularly T cells and NK cells, in responding to stress and inflammation during DW healing, and underscored the therapeutic potential of ESWT. Enrichment analysis revealed that T cells were strongly associated with immune-regulatory pathways, including regulation of interleukin-12 production and granulocyte migration, indicating their role in modulating cytokine signaling and coordinating immune cell recruitment. B cells were primarily enriched in detoxification- and oxidative stress–related processes, such as cellular detoxification, oxidant detoxification, and response to toxic substances, suggesting potential involvement in maintaining redox balance within the wound microenvironment. DCs were associated with apoptotic and differentiation-related pathways, including extrinsic apoptotic signaling and regulation of erythrocyte differentiation, highlighting their role in antigen presentation and immune regulation. NK cells were enriched in mitochondrial and apoptosis-regulatory processes, particularly negative regulation of mitochondrial membrane permeability, reflecting their contribution to cytotoxicity and immune surveillance.

To investigate how ESWT modulated immune cell interactions in the DW microenvironment, we performed cell–cell communication analysis across the immune cells (Figure 10D). The number of inferred interactions showed a clear increase following ESWT treatment, with ESWT7d exhibiting the highest value of 589, compared to control (323) and DW (405). This suggested that ESWT treatment enhanced the overall cellular communication, especially at the 7-day time point. The interaction strength also followed a similar trend, with ESWT7d showing the highest interaction strength (13.841), significantly higher than the control (6.024) and DW (8.49) groups. Notably, while the number of inferred interactions in ESWT14d was lower than in ESWT7d, it remained higher than in the control and DW groups, indicating that ESWT treatment continued to support stronger cell-to-cell interactions over time, particularly in the early stages (Figure 10E). These results underscored that ESWT not only enhanced the quantity of cellular interactions but also improves the quality of these interactions, which likely contributed to better immune activation and tissue repair in DWs.

The scatter plots depicted the incoming and outgoing interaction strength for various immune cell types across different groups (Figure 10F). In the control group, Effector_CD8 cells exhibited a relatively low outgoing interaction strength but high incoming interaction strength, indicating that they received more signals than they send. T cells, on the other hand, displayed high outgoing interaction strength, signifying their active role in cell signaling. In the DW group, the interaction profile is altered, with NK cells showing a notable increased in outgoing interaction strength, suggesting a stronger immune response in diabetic wounds. However, the Effector_CD8 cells remain at the lower end of the outgoing strength, indicating impaired activation in this group. After ESWT treatment, particularly at ESWT7d, NK cells and cDC1 exhibited significantly higher outgoing interaction strength compared to the DW group, reflecting a restoration of immune function. Treg also showed increased outgoing interactions, which suggested enhanced immune modulation and communication after ESWT. Overall, these results demonstrated that ESWT enhanced immune cell communication, particularly the activation of cDC1 and NK cells, which was crucial for efficient tissue repair in DWs.

Then we analyzed the differences in cell communication pathways across different treatment groups (Figure 10G). In the ESWT7d group, several signaling pathways were upregulated, including interactions between Sema4a - Cd72, Cd80 - Cd274, and App - Cd74, indicating enhanced immune cell activation and communication following ESWT treatment. These upregulated interactions were primarily seen in T cells, NK cells, and DC cells, suggesting that ESWT promoted immune activation and enhanced the communication between key immune cell populations. Notably, the pathways involving Spp1 and Thbs1 were significantly upregulated, which were associated with tissue remodeling and inflammation regulation, further supporting the role of ESWT in promoting tissue repair in DWs. In contrast, the DW group showed downregulated signaling in several important pathways, such as Pvr - Cd226, Cd70 - Cd27, and Ccl3 - Ccr5, highlighting impaired immune cell communication in diabetic wounds. The downregulation of these pathways, particularly in NK cells and T cells, suggested that the immune response in DW was weakened, potentially contributing to the delayed healing and chronic inflammation observed in DWs. The Ccl5 - Ccr5 interaction was also downregulated in DW, which was associated with a reduced ability to recruit immune cells to the wound site, further impeding tissue repair.

**FIGURE 11:**
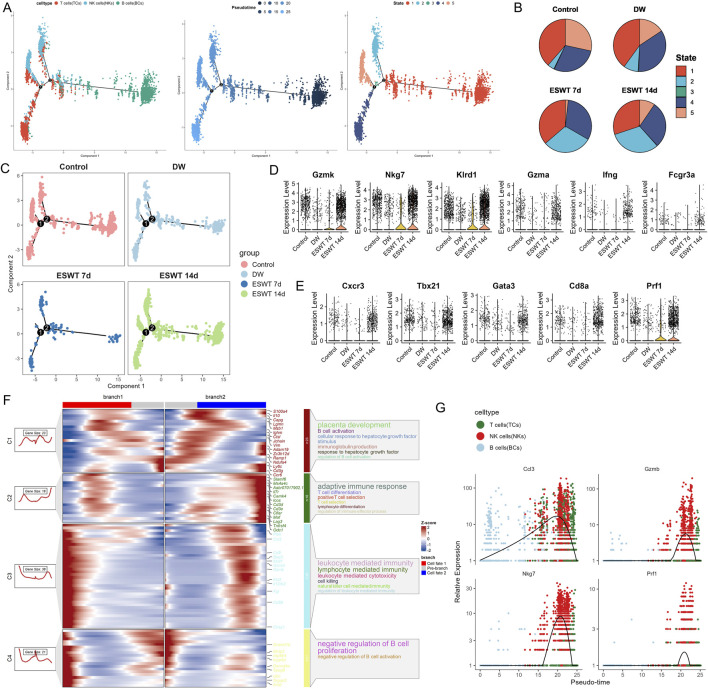
ESWT promoted immune activation and enhanced cytotoxic programs in DWs. **(A)** Pseudotime trajectory analysis of TCs, NKs, and BCs. Cells were ordered along pseudotime (middle) and grouped into distinct states (right), revealing dynamic immune transitions. **(B)** Pie charts illustrating pseudotime state distributions across Control, DW, ESWT 7d, and ESWT 14d groups, showing that ESWT redirected immune cells toward advanced functional states. **(C)** Pseudotime distribution plots across groups, showing that ESWT remodeled immune cell trajectories compared with DW. **(D)** Violin plots of cell cytotoxicity-related genes (Gzmk, Nkg7, Klrd1, Gzma, Ifng, Fcgr3a), demonstrating upregulation under ESWT. **(E)** Violin plots of T cell transcription factors and effector genes (Cxcr3, Tbx21, Gata3, Cd8a, Prf1), showing increased expression in ESWT groups. **(F)** Branched heatmap of pseudotime-dependent gene expression modules. Cluster 1 genes were enriched in growth factor signaling, cluster 2 in adaptive immune response, cluster 3 in lymphocyte-mediated cytotoxicity, and cluster 4 in B cell regulation, highlighting ESWT-driven immune reprogramming. **(G)** Smoothed pseudotime expression of representative cytotoxic and effector genes (Cd3, Gzmb, Nkg7, Prf1), confirming progressive activation of lymphocyte cytotoxic programs under ESWT treatment.

While T cells, B cells, and NK cells belong to distinct lymphoid lineages, they collectively participate in the adaptive and innate immune responses during wound healing. Therefore, we examined their activation dynamics within the lymphoid compartment to capture ESWT-induced immune coordination rather than developmental transitions. Using Monocle2, we explored the dynamic activation trajectories of immune cells to reveal how ESWT influenced their functional states during diabetic wound healing. The presented plots visualized pseudotime trajectories and cell state distributions across different groups. As shown in Figure 11A, the distribution of immune cells—T cells, NK cells, and B cells was plotted along the pseudotime trajectory. The trajectory highlighted different stages of immune cell activation and differentiation, with T cells and NK cells showing distinct progression patterns. The pie charts summarized the distribution of cell states across the different groups (Figure 11B). In the control group, there was a more balanced distribution across the cell states, while in DW, a larger proportion of cells are stuck in early states, reflecting impaired immune responses. In ESWT7d and ESWT14d, there was a noticeable shift towards more advanced states, particularly in T cells and NK cells, suggesting that ESWT accelerated immune cell activation and enhanced the transition through the various cell states, ultimately promoting a more efficient immune response and wound healing process. Immune cells in control group showed a clear separation of cell states, with T cells and NK cells progressing through distinct states, indicating a more organized immune response (Figure 11C). In contrast, the DW group displayed a less defined trajectory, suggesting impaired immune responses in DWs. After ESWT treatment, the pseudotime trajectories showed a shift towards more organized and dynamic immune activation. The ESWT7d group, in particular, showed a more robust progression through the cell states, indicating that ESWT promoted immune activation and enhanced cell state transitions, especially in T cells and NK cells. The ESWT14d group continued to show improved immune activation, but with some stabilization of the trajectories compared to ESWT7d, suggesting the potential long-term effects of ESWT in modulating immune responses.

The violin plots presented the expression levels of key immune-related genes across different groups (Figures 11D,E). These genes were associated with various immune cell functions and activation states, particularly in T cells, NK cells, and cytotoxic T cells. These results suggested that ESWT treatment significantly enhanced the activation, differentiation, and cytotoxic function of immune cells, particularly T cells and NK cells, which were essential for efficient wound healing in diabetic conditions. The observed expression changes in these immune-related genes highlighted the immune-modulatory role of ESWT in promoting tissue repair and reducing inflammation in diabetic wounds. The results from the Monocle2 pseudotime analysis revealed key insights into the immune cell differentiation and activation processes in response to ESWT treatment in DW healing. The heatmap displayed the expression dynamics of key immune-related genes across different pseudotime trajectories, grouped into four clusters, with each cluster representing distinct patterns of gene expression during immune cell activation and differentiation (Figure 11F). The genes associated with adaptive immune response showed significant expression changes across pseudotime, highlighting the immune system’s transition from an initial response to a more activated state. In particular, cluster C2 was enriched for genes involved in adaptive immune response and T cell differentiation, including Cd3e, Camk4, and Tbx21. Cluster C3 showed a strong association with leukocyte-mediated immunity, with markers such as Gzmb and Gzm being upregulated, suggesting the activation of cytotoxic immune responses. The expression patterns of Gzmb and Nkg7 peaked at later stages of pseudotime, indicating that ESWT might have enhanced cytotoxic T cell and NK cell functions in promoting wound healing. Relative expression of key genes across pseudotime was shown in Figure 11G. Genes like Ccl3, Gzmb, and Nkg7 exhibited sharp peaks at specific pseudotime points, which correlated with immune activation. On the other hand, genes like Prf1 showed a more gradual increase in expression, suggesting a delayed but sustained activation, which was crucial for long-term tissue repair and immune modulation. Overall, these findings indicated that ESWT facilitated immune cell activation, enhanced cytotoxic responses, and drove immune differentiation in the wound microenvironment, thus potentially improving healing outcomes in diabetic wounds by modulating both the innate and adaptive immune responses.

## Discussion

Delayed wound healing in diabetes remains a major clinical challenge, characterized by persistent inflammation, impaired angiogenesis, and defective extracellular matrix remodeling. scRNA-seq provides an unprecedented resolution to dissect the complex cellular landscape of diabetic wounds. Traditional bulk transcriptomic approaches often mask the heterogeneity of different cell types within the wound microenvironment, limiting our understanding of the cellular and molecular mechanisms driving impaired healing. By contrast, scRNA-seq enables the precise identification of distinct cell subpopulations and their dynamic transcriptional states, offering crucial insights into intercellular communication, lineage differentiation, and regulatory networks (Zhao et al., 2025). In the context of diabetic foot ulcers (DFU), this technology is particularly powerful for uncovering disease-specific endothelial cell subsets and their molecular signatures that govern inflammation and angiogenesis (Lu et al., 2023; Dong et al., 2024).

In this study, we demonstrated that ESWT significantly accelerated wound closure and enhanced tissue regeneration in diabetic rats. Macroscopic observations and histological analyses confirmed that ESWT markedly improved re-epithelialization and collagen deposition, suggesting that ESWT effectively promotes both the speed and quality of diabetic wound repair. The improvement in healing observed after ESWT may be attributed to its ability to activate mechanotransduction signaling pathways, enhance local blood flow, and stimulate angiogenic and reparative processes. Beyond the phenotypic improvement, we further generated a comprehensive single-cell transcriptomic atlas of cell subpopulations in DWs and revealed how ESWT dynamically reshaped cellular heterogeneity during wound healing. Our results demonstrated that ESWT not only altered the relative abundance of cellular subsets but also reprogrammed their transcriptional states and trajectories toward pro-repair phenotypes.

Macrophages play a pivotal role in the pathophysiology of DW healing (Sharifiaghdam et al., 2022). As key regulators of innate immunity and tissue remodeling (Louiselle et al., 2021), macrophages orchestrate inflammatory responses (Tang et al., 2023), clearance of debris (Guan et al., 2025), angiogenesis (Martin and Gurevich, 2021), and extracellular matrix turnover (Yu et al., 2024). In the context of diabetes, however, their function becomes dysregulated, leading to a sustained pro-inflammatory state that impairs the normal progression of wound repair. Previous studies have shown that DWs were characterized by an imbalance between pro-inflammatory M1-like macrophages and reparative M2-like macrophages, with excessive persistence of M1 macrophages driving chronic inflammation, delayed re-epithelialization, and defective vascularization (Al, 2022). Our single-cell analysis further reinforces this paradigm by demonstrating that macrophage heterogeneity is profoundly altered in DW tissues. Distinct subpopulations, including Il18bp^hi^ and Pglyrp1^hi^ Mac, displayed pro-inflammatory gene signatures, whereas Clec10a-positive macrophages exhibited transcriptional programs associated with immune resolution and tissue repair. These findings underscore that macrophages are not a uniform population but instead consist of diverse functional states that collectively dictate wound outcomes. Importantly, ESWT treatment appeared to reprogram macrophage composition and polarization, promoting the expansion of reparative subsets while reducing the dominance of pro-inflammatory states. This highlights macrophages as both drivers of diabetic wound pathology and critical therapeutic targets for interventions aimed at restoring the balance between inflammation and repair.

Fibroblasts are indispensable for wound healing, contributing to ECM deposition, tissue remodeling, and paracrine signaling that regulates immune and vascular responses (Voza et al., 2024). In DWs, however, fibroblast function is impaired, leading to defective ECM synthesis, persistent inflammation, and inadequate angiogenesis (Liu et al., 2022). Our single-cell analysis uncovered marked fibroblast heterogeneity in DW tissues and revealed that ESWT profoundly reshaped fibroblast subpopulations, transcriptional programs, and differentiation trajectories to favor wound repair. We identified nine fibroblast subclusters with distinct specializations. Angptl1^hi^ and Mmp13^hi^ Fbs, Fbsenriched in ECM remodeling pathways, were significantly restored by ESWT, particularly at 14 days, suggesting their role as reparative fibroblast phenotypes. Conversely, Clec4a3^hi^, Ccl6^hi^, and Gdf15^hi^ Fbs, associated with immune regulation and inflammation, predominated in DWs but were reduced after ESWT, indicating a rebalancing toward pro-healing states. Intersection analysis of ESWT-responsive genes further highlighted fibroblast-mediated repair programs, including ECM organization, cell adhesion, cytoskeletal regulation, and PI3K–AKT/NF-κB signaling. Trajectory analysis confirmed that ESWT accelerated the transition of fibroblasts toward late reparative states, restoring patterns closer to healthy controls. Importantly, ECM-related genes were upregulated, while inflammatory mediators were suppressed, underscoring a shift from pro-inflammatory to regenerative phenotypes. These findings highlight fibroblasts as key therapeutic targets of ESWT and suggest that modulation of fibroblast diversity is an important mechanism underlying ESWT-mediated wound healing.

Neutrophils are among the earliest immune cells recruited to wound sites, where they contribute to microbial defense and debris clearance (de Oliv et al., 2016). However, in DWs, their persistent activation sustains inflammation, disrupts tissue repair, and delays wound closure (Zhu et al., 2023). Our single-cell transcriptomic analysis revealed three transcriptionally distinct neutrophil subsets—S100a8^hi^, Thbs1^hi^, and Gbp2^hi^—that displayed dynamic changes in response to ESWT. In DWs, neutrophils were dominated by S100a8^hi^ clusters, enriched in chemotaxis and NETosis-related programs, consistent with a pro-inflammatory state. ESWT treatment rebalanced this composition by inducing an early expansion of Thbs1^hi^ neutrophils, associated with hypoxia response and angiogenesis, followed by an enrichment of Gbp2^hi^ interferon-responsive neutrophils at later stages. This temporal shift suggests that ESWT accelerates the transition from inflammatory to reparative neutrophil phenotypes. Trajectory and functional analyses further supported this model, showing that ESWT reduced pro-inflammatory gene expression and NETosis potential, while modestly enhancing reparative genes. These findings highlight neutrophils as critical regulators of the inflammatory–reparative continuum in DWs and suggest that ESWT promotes wound healing in part by reshaping neutrophil heterogeneity, dampening pathological inflammation, and enhancing pro-angiogenic and reparative functions.

KCs are essential for wound re-epithelialization and barrier restoration, but in DWs their differentiation is impaired and they adopt a persistently pro-inflammatory phenotype (Song et al., 2025). Our single-cell analysis revealed profound KC heterogeneity and demonstrated that ESWT effectively reshaped KC subpopulation dynamics and transcriptional programs. We identified six KC subtypes with distinct roles, including barrier-forming (Krtdap^hi^, Tgm5^hi^), immune-modulatory (Igfbp2^hi^, Nos2^hi^), stress-responsive (Mt4^hi^), and cytotoxic (Gzmk^hi^) populations. In DWs, most subsets were markedly reduced, consistent with impaired epidermal renewal. ESWT treatment restored multiple populations, particularly Krtdap^hi^ and Tgm5^hi^ subsets, suggesting reactivation of differentiation and barrier repair. Importantly, ESWT also suppressed the expansion of Nos2^hi^ inflammatory keratinocytes, indicating attenuation of pathological inflammation. Trajectory analysis further revealed that DW KCs were biased toward late-stage states, reflecting delayed or dysregulated differentiation. ESWT shifted trajectories toward earlier and intermediate states, consistent with accelerated renewal and recovery of functional epidermis. Correspondingly, inflammatory mediators were downregulated, while epidermal differentiation markers were restored after ESWT. These changes highlight a coordinated transition from immune activation to structural repair in keratinocytes. Collectively, these findings indicate that ESWT promotes diabetic wound healing not only by suppressing keratinocyte-driven inflammation but also by re-establishing differentiation programs required for barrier integrity. This dual effect underscores keratinocytes as both contributors to DW pathology and key cellular targets through which ESWT exerts its reparative efficacy.

ECs are central to angiogenesis and vascular remodeling, both of which are essential for wound repair (Citrin et al., 2025). In DWs, EC dysfunction contributes to impaired vascularization, persistent inflammation, and delayed healing (Pang et al., 2024). Our single-cell analysis revealed profound EC heterogeneity, with six transcriptionally distinct subclusters exhibiting diverse biological functions. Importantly, ESWT significantly reshaped this landscape, shifting ECs from inflammatory and DW-associated states toward pro-angiogenic and reparative phenotypes. We found that DW tissues were enriched in Ereg^hi^ and Lhx2^hi^ ECs, populations linked to inflammatory signaling and dysregulated vascular responses. In contrast, ESWT markedly expanded Cadps2^hi^ and Prox1^hi^ ECs, subsets associated with angiogenesis and lymphangiogenesis, suggesting that ESWT reprograms ECs toward regenerative states. Functional enrichment and AUCell analyses further confirmed this transition, as ESWT enhanced angiogenic signatures while reducing inflammatory activity. Pseudotime trajectory analysis supported these findings by showing that DW ECs were largely confined to states dominated by inflammatory subclusters, whereas ESWT promoted a redistribution toward Branch 1 trajectories, characterized by elevated expression of angiogenesis-related genes and survival markers, alongside suppression of pro-apoptotic and inflammatory genes. BEAM analysis further highlighted the divergence between reparative and inflammatory programs, reinforcing the notion that ESWT fosters a pro-angiogenic EC trajectory while attenuating maladaptive immune responses. Collectively, these results demonstrate that ESWT exerts profound effects on EC heterogeneity, selectively enriching subclusters with high angiogenic potential and redirecting developmental trajectories toward pro-repair states. By enhancing vascular integrity and promoting angiogenesis while suppressing apoptosis and inflammation, ESWT restores EC functionality in DWs and provides a cellular basis for its therapeutic efficacy in chronic wound healing.

SMCs play a crucial role in vascular homeostasis and wound healing by maintaining vascular tone, supporting ECM remodeling, and stabilizing neovessels (Smith and Rai, 2024). In DWs, however, SMCs lose their contractile phenotype and adopt a pro-inflammatory state, thereby compromising vascular integrity and delaying repair. Our single-cell analysis revealed marked heterogeneity among SMCs, identifying six transcriptionally distinct subtypes with diverse functional programs. Importantly, ESWT markedly reprogrammed the SMC landscape, shifting cells from inflammatory and proliferative states toward reparative, contractile phenotypes. We found that DW tissues were enriched in Trem1^hi^ SMCs, which exhibited strong associations with granulocyte and leukocyte chemotaxis, consistent with an inflammatory-activated profile. In contrast, Sncg^hi^ and Postn^hi^ SMCs, subsets linked to ECM organization, vessel stabilization, and structural remodeling, were significantly restored by ESWT, particularly at 14 days. Functional scoring further supported these findings, as ESWT suppressed the inflammation phenotype while restoring contractile gene expression, suggesting a recovery of vascular-supportive functions. Trajectory analysis provided additional insights into SMC plasticity. In DWs, SMCs were biased toward early, inflammation-dominated states, whereas ESWT promoted progression along reparative branches enriched in cytoskeletal remodeling, actomyosin structure organization, and calcium signaling pathways. BEAM analysis confirmed that ESWT reduced the dominance of immune-oriented trajectories while enhancing repair-oriented ones, underscoring its role in redirecting SMC fate toward vascular stabilization and tissue regeneration. Collectively, these findings indicate that ESWT alleviates the inflammatory activation of SMCs and promotes their re-differentiation into contractile, vascular-supportive phenotypes. By restoring SMC heterogeneity and function, ESWT contributes to improved vascular remodeling, ECM organization, and overall wound healing in DWs.

Immune cells are critical regulators of DW healing, coordinating inflammation resolution, pathogen clearance, and tissue repair (Deng et al., 2023). In chronic DWs, however, the immune response is often dysregulated, leading to persistent inflammation and impaired repair (Moura et al., 2019). Our single-cell analysis revealed that ESWT profoundly reshaped the immune landscape, modulating the distribution, activation states, and intercellular communication of T cells, B cells, NK cells, and DCs. In untreated DWs, immune cell populations were markedly imbalanced, with reduced DC and lymphocyte representation and impaired T cell and NK cell activation. These findings are consistent with previous reports showing that defective antigen presentation and cytotoxic function are hallmarks of chronic non-healing wounds. ESWT progressively restored immune cell proportions, particularly enhancing DCs and NK cells, thereby re-establishing a balanced immune microenvironment. Cell–cell communication analysis highlighted that ESWT not only increased the number of interactions but also enhanced their functional quality. ESWT 7d showed the most robust communication network, characterized by enriched pathways involving Sema4a–Cd72, CD80–CD274, and Spp1–Thbs1, which are associated with immune activation, tissue remodeling, and inflammatory resolution. In contrast, DWs displayed downregulated signaling in Pvr–Cd226, Cd70–Cd27, and Ccl5–Ccr5, reflecting weakened immune crosstalk. Notably, ESWT boosted outgoing signaling strength from NK cells, cDC1, and Treg cells, underscoring its role in promoting cytotoxic activity and immunomodulation. Trajectory analysis provided further mechanistic insights. In DWs, immune cells were stalled in early states, reflecting impaired activation. ESWT facilitated the progression of T and NK cells through more advanced pseudotime states, indicating accelerated immune activation and differentiation. Gene expression dynamics along pseudotime revealed upregulation of cytotoxic markers (Gzmb, Nkg7, Prf1) and adaptive immune regulators (Cd3e, Tbx21, Camk4) after ESWT, supporting enhanced cytotoxic T cell and NK cell functions. Importantly, ESWT also reduced excessive expression of inflammatory mediators, suggesting a shift toward balanced immune activation rather than uncontrolled inflammation. Collectively, our findings indicate that ESWT exerts broad immunomodulatory effects in DWs, restoring immune cell composition, enhancing adaptive and cytotoxic responses, and strengthening cell–cell communication networks. By promoting DC and NK cell activation and supporting T cell differentiation, ESWT reprograms the immune microenvironment from a dysfunctional, inflammation-skewed state to a reparative, coordinated immune response. This highlights immune cells as both biomarkers of therapeutic efficacy and key mediators of ESWT-driven wound healing.

Nevertheless, limitations should be acknowledged. While scRNA-seq offers high-resolution insights, functional validation *in vivo* and in clinical cohorts is needed to establish causality and translational relevance. Moreover, the long-term impact of ESWT on systemic immunity and metabolic regulation remains to be elucidated.

In summary, our single-cell transcriptomic analysis provides a comprehensive atlas of the cellular and molecular mechanisms underlying ESWT-mediated DW healing. We demonstrate that ESWT dynamically reprograms the wound microenvironment by modulating the heterogeneity and functional states of immune, stromal, and vascular cell populations. Specifically, ESWT attenuates pathological inflammation, restores keratinocyte differentiation, rebalances macrophage and fibroblast subpopulations, reshapes neutrophil phenotypes, and promotes endothelial and smooth muscle cell programs that support angiogenesis and vascular stabilization. These coordinated cellular transitions converge to accelerate tissue repair and barrier restoration. Our findings highlight the multifaceted immunomodulatory and pro-angiogenic effects of ESWT, providing mechanistic insights into its clinical efficacy in chronic wound management. Importantly, the identification of key reparative subpopulations suggests potential cellular biomarkers and therapeutic targets that may guide precision application of ESWT or combined therapeutic strategies.

## Conclusion

In conclusion, our study provides a comprehensive single-cell transcriptomic atlas of DWs and reveals the cellular and molecular mechanisms underlying the therapeutic effects of ESWT. These findings highlight the multicellular and pro-angiogenic mechanisms by which ESWT accelerates DW healing and underscore its value as a non-invasive therapeutic strategy. By identifying key reparative cell subsets and regulatory pathways, our work not only advances the biological understanding of ESWT but also provides a foundation for the development of targeted interventions to improve outcomes in patients with chronic, non-healing wounds.

## Data Availability

The data presented in the study are deposited in the China National Center for Bioinformation database, accession number PRJCA050405.
