# Correction: Single-cell transcriptome analysis profiles cellular dynamics and transcriptional changes in diabetic wound tissues following ESWT treatment

**DOI:** 10.3389/fphys.2026.1772233

**Published:** 2026-02-09

**Authors:** Dongyu Li, Yu Wang, Yunlong Wang, Changhai Shao, Lei Wang, Shijie Xin, Yuewen Ma

**Affiliations:** 1 Department of VIP In-Patient Ward, The First Hospital of China Medical University, Shenyang, Liaoning, China; 2 Department of Rehabilitation, The First Hospital of China Medical University, Shenyang, Liaoning, China; 3 Department of Thoracic Surgery, Angang General Hospital, Anshan, Liaoning, China; 4 Department of Vascular and Thyroid Surgery, The First Hospital of China Medical University, Shenyang, Liaoning, China

**Keywords:** diabetic wound, extracorporeal shock wave therapy, single cell RNA sequencing, endothelial cells, microenvironment remodeling

In the published article there were errors in the author list, affiliations and one part of the text.

**Affiliation** “Department of Rehabilitation, The First Hospital of China Medical University, Shenyang, Liaoning, China” was omitted for author Dongyu Li. This affiliation has now been added for author Dongyu Li.

**Author** Dongyu Li was erroneously assigned to affiliation “Department of Thoracic Surgery, Angang General Hospital, Anshan, Liaoning province, China”. This affiliation has now been removed for author Dongyu Li.

Author Changhai Shao was erroneously spelled as Changhai Sihao.

A correction has been made to the section **Materials and methods**, *Establishment of a Streptozotocin(STZ)-Induced DW Model and ESWT treatment:* “Shock waves were applied at an energy flux density of 0.12 mJ/mm^2^ with a frequency of 3 Hz, delivering 100 pulses per centimeter of wound length, as previously described.”

The original article has been updated.

